# A high-quality genome and comparison of short- versus long-read transcriptome of the palaearctic duck *Aythya fuligula* (tufted duck)

**DOI:** 10.1093/gigascience/giab081

**Published:** 2021-12-20

**Authors:** Ralf C Mueller, Patrik Ellström, Kerstin Howe, Marcela Uliano-Silva, Richard I Kuo, Katarzyna Miedzinska, Amanda Warr, Olivier Fedrigo, Bettina Haase, Jacquelyn Mountcastle, William Chow, James Torrance, Jonathan M D Wood, Josef D Järhult, Mahmoud M Naguib, Björn Olsen, Erich D Jarvis, Jacqueline Smith, Lél Eöry, Robert H S Kraus

**Affiliations:** Department of Migration, Max Planck Institute of Animal Behavior, Radolfzell, 78315, Germany; Department of Biology, University of Konstanz, Konstanz, 78457, Germany; Department of Medical Sciences, Zoonosis Science Center, Uppsala University, Uppsala, SE-75185, Sweden; Tree of Life, Wellcome Sanger Institute, Cambridge CB10 1SA, UK; Tree of Life, Wellcome Sanger Institute, Cambridge CB10 1SA, UK; The Roslin Institute and Royal (Dick) School of Veterinary Studies, University of Edinburgh, Easter Bush, Midlothian EH25 9RG, UK; The Roslin Institute and Royal (Dick) School of Veterinary Studies, University of Edinburgh, Easter Bush, Midlothian EH25 9RG, UK; The Roslin Institute and Royal (Dick) School of Veterinary Studies, University of Edinburgh, Easter Bush, Midlothian EH25 9RG, UK; Vertebrate Genome Laboratory, The Rockefeller University, New York, 10065, NY; Vertebrate Genome Laboratory, The Rockefeller University, New York, 10065, NY; Vertebrate Genome Laboratory, The Rockefeller University, New York, 10065, NY; Tree of Life, Wellcome Sanger Institute, Cambridge CB10 1SA, UK; Tree of Life, Wellcome Sanger Institute, Cambridge CB10 1SA, UK; Tree of Life, Wellcome Sanger Institute, Cambridge CB10 1SA, UK; Department of Medical Sciences, Zoonosis Science Center, Uppsala University, Uppsala, SE-75185, Sweden; Department of Medical Biochemistry and Microbiology, Zoonosis Science Center, Uppsala University, Uppsala, 75237, Sweden; Department of Medical Sciences, Zoonosis Science Center, Uppsala University, Uppsala, SE-75185, Sweden; Vertebrate Genome Laboratory and HHMI, The Rockefeller University, New York, 10065, NY; The Roslin Institute and Royal (Dick) School of Veterinary Studies, University of Edinburgh, Easter Bush, Midlothian EH25 9RG, UK; The Roslin Institute and Royal (Dick) School of Veterinary Studies, University of Edinburgh, Easter Bush, Midlothian EH25 9RG, UK; Department of Migration, Max Planck Institute of Animal Behavior, Radolfzell, 78315, Germany; Department of Biology, University of Konstanz, Konstanz, 78457, Germany

**Keywords:** tufted duck, Aythya fuligula, genome annotation, transcriptome sequencing, Vertebrate Genomes Project, Iso-Seq, Pacific Biosciences, RNA sequencing, small RNA

## Abstract

**Background:**

The tufted duck is a non-model organism that experiences high mortality in highly pathogenic avian influenza outbreaks. It belongs to the same bird family (Anatidae) as the mallard, one of the best-studied natural hosts of low-pathogenic avian influenza viruses. Studies in non-model bird species are crucial to disentangle the role of the host response in avian influenza virus infection in the natural reservoir. Such endeavour requires a high-quality genome assembly and transcriptome.

**Findings:**

This study presents the first high-quality, chromosome-level reference genome assembly of the tufted duck using the Vertebrate Genomes Project pipeline. We sequenced RNA (complementary DNA) from brain, ileum, lung, ovary, spleen, and testis using Illumina short-read and Pacific Biosciences long-read sequencing platforms, which were used for annotation. We found 34 autosomes plus Z and W sex chromosomes in the curated genome assembly, with 99.6% of the sequence assigned to chromosomes. Functional annotation revealed 14,099 protein-coding genes that generate 111,934 transcripts, which implies a mean of 7.9 isoforms per gene. We also identified 246 small RNA families.

**Conclusions:**

This annotated genome contributes to continuing research into the host response in avian influenza virus infections in a natural reservoir. Our findings from a comparison between short-read and long-read reference transcriptomics contribute to a deeper understanding of these competing options. In this study, both technologies complemented each other. We expect this annotation to be a foundation for further comparative and evolutionary genomic studies, including many waterfowl relatives with differing susceptibilities to avian influenza viruses.

## Background

The tufted duck (*Aythya fuligula*, NCBI:txid219594) is a non-model organism that has received attention because of its role in the zoonotic ecology of avian influenza A viruses (AIVs). As a member of the Anatidae family of ducks, it is closely related to the mallard (*Anas platyrhynchos*), the primary natural host of AIV [1–5]. The *Aythya* and *Anas* genera shared a most recent common ancestor ∼5 million years ago [6]. However, in contrast to mallards, which carry AIV largely asymptomatically, tufted ducks are less commonly infected with low pathogenic AIV (LPAIV) (see [7] for an updated review on LPAIV infections in tufted ducks) but experience high mortality in outbreaks of highly pathogenic AIV (HPAIV) [8–11]. The tufted duck is a diving duck with a breeding range throughout northern Eurasia, where it is largely a seasonal short-distance migrant. Although it generally feeds in deeper waters than mallards and other dabbling ducks, it generally shares its habitat and roosting areas with these and many other waterfowl species, even leading to a high rate of interspecific hybridization [12,13]. Hence, given a frequent exposure to AIVs circulating in such habitats, the differences in susceptibility to—and outcome of—AIV infection between these species are likely related to genetic differences affecting, e.g., receptor expression or host response. For example, studies of virus attachment patterns to tissue samples have shown that many AIV subtypes bind less abundantly to intestinal epithelial cells of tufted ducks compared to mallards [7,14]. In addition, the resistance of mallards against severe HPAIV infection has been partially attributed to the presence of the *RIG-I*gene and its strong interferon response, in contrast to chickens, which lack this gene and develop severe disease upon HPAIV infection [15]. Future studies in non-model bird species such as the tufted duck are important to disentangle the role of the host response and other genetic factors in AIV infection and aid in our understanding of the zoonotic ecology of AIV in the natural reservoir. A prerequisite for such studies is a well-assembled and annotated genome and transcriptome [16].

Developments in omic sequencing technologies over the past 2 decades have revolutionized biology. Instead of studying single genes and their products, whole genomes and transcriptomes can now be readily sequenced and assembled at a lower cost than before. Massive parallelization and high throughput in next-generation sequencing (NGS) have decreased sequencing costs and ultimately increased sequencing depth [17]. This allows for whole-genome sequencing of any species and opens up new possibilities for in-depth studies related to infection biology and host response to external stressors beyond model species in which a rich genetic toolbox can be deployed [18,19]. Non-model species are frequently understudied, yet they are exposed to environmental stressors such as infectious diseases, which they can transmit to livestock and humans [20]. Approximately 70% of human infectious diseases are zoonoses [21,22]. An in-depth understanding of a pathogen’s zoonotic ecology in the animal reservoir is important to prevent human infections. This, in turn, requires studies of host-pathogen interactions at the genetic level. NGS helps bridge the gap between model and non-model species [23], and with third-generation long-read–based sequencing, high-quality reference genomes are now also available for non-model organisms, as in the Vertebrate Genomes Project (VGP) [24]. This is supplemented by affordable long-read RNA sequencing, making *de novo* assembled transcriptomes unnecessary [25].

Transcriptome annotation of the genome used to be constrained to either low-throughput and costly complementary DNA (cDNA) cloning or Illumina’s high-throughput short-read RNA sequencing (RNA-Seq). High-quality short-read RNA sequencing combined with a reference genome allows for a reasonable transcriptome reconstruction. However, there are some caveats: Owing to alternative splicing, a single gene can have multiple alternative variants (isoforms) and as a consequence can be translated into proteins with different functions [26,27]. Illumina short reads need to be assembled into transcripts, which can lead to incompleteness and errors in transcript model reconstruction. This biases the correct inference of isoforms and thus misses the transcriptome’s complexity [26,28]. Furthermore, because short-read sequencing is also limited in GC-rich regions and regions of low sequence complexity [29], not all transcripts are recovered. In contrast, in full-length transcript isoform sequencing (e.g., Pacific Biosciences [PacBio] Iso-Seq) of messenger RNA, the single direct reads from 5′ to 3′ usually do not need to be assembled and thus prevent assembly ambiguities, which facilitates the detection of novel isoforms [30, 31] and accurate reconstruction of transcript structure [29]. This third-generation transcript sequencing technology also allows for a much more detailed functional annotation. It is crucial to recover as many isoforms as possible for functional studies [32–34]. For example, the immunome comprises a set of genes associated with immune processes and is a heavily complex portion of the genome [25,35]. Thus, the benefits of third-generation sequencing must be exploited to their fullest potential to facilitate studies in bird immunogenomics [36] and improve databases such as the Avian Immunome DB for comparative analyses in non-model bird species [37].

## Data Description

This study presents the first high-quality, chromosome-level reference genome assembly of the tufted duck using the VGP pipeline [24] followed by manual curation [38], which we annotated with short- and long-read transcripts from 6 different tissues, and short reads from small RNAs from the same tissues. We further contrasted different sequencing technologies and bioinformatics pipelines, and tissue-specific comparisons of expressed genes. This annotation serves as a foundation for further comparative and evolutionary genomics, and gene expression experiments in the tufted duck and its many waterfowl relatives with different susceptibilities to AIV [36,39].

## Analyses

### Reference genome assembly

**Figure 1:**
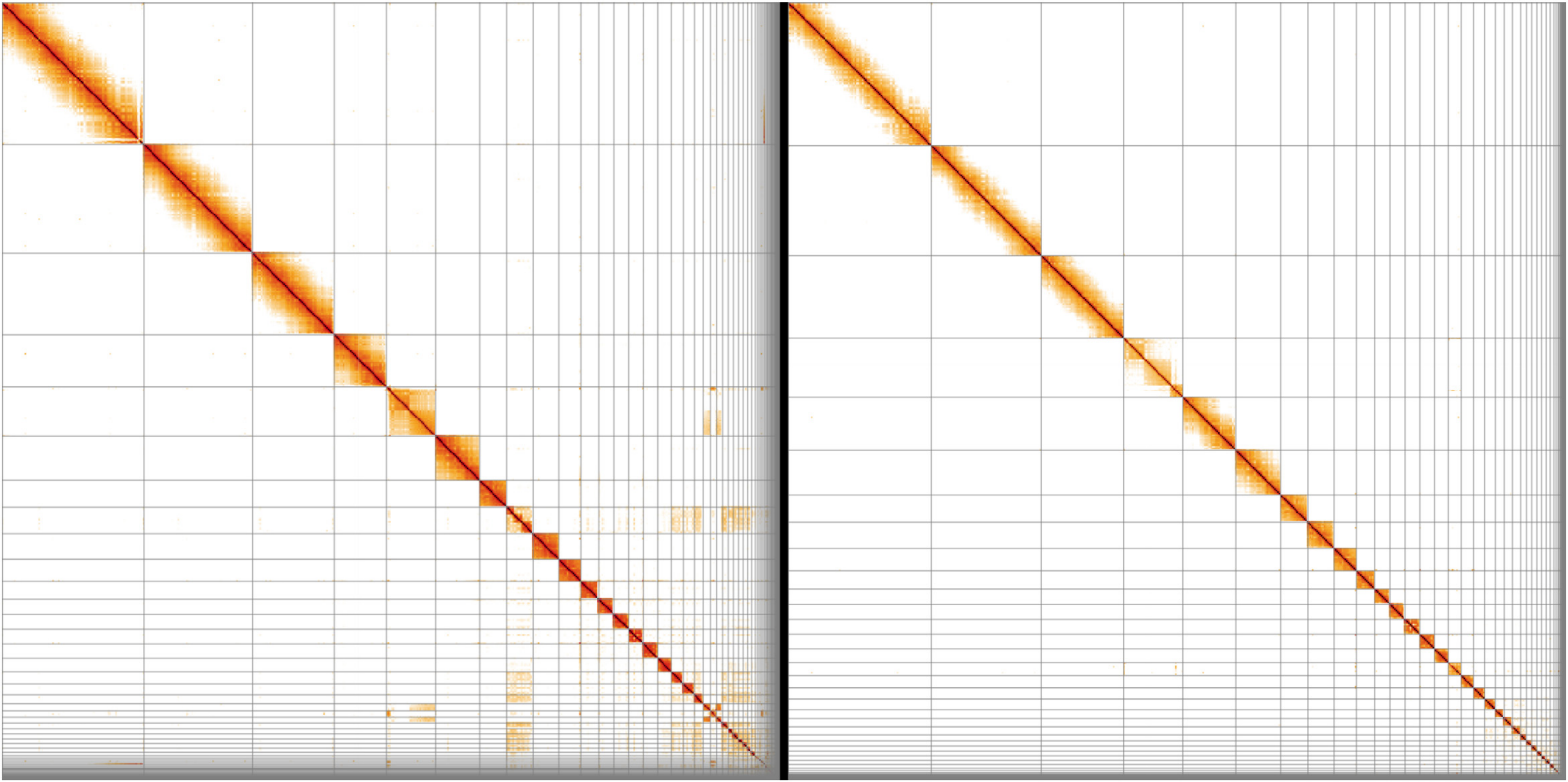
HiGlass Hi-C 2D maps of the tufted duck genome assembly before (left) and after (right) manual curation. Off-diagonal hits indicate missing joins, which have been corrected during curation. Broken patterns within scaffolds (e.g., at the end of the first scaffold before curation) can indicate intra-scaffold misassemblies, which were also addressed during curation. They can, however, also be features of the respective chromosome, as in the fourth post-curation scaffold, the structure of which was corrected and asserted during curation.

The reference genome was constructed according to VGP’s 6.7.P2.Q40.C99 metric standards [24]. This includes contig NG50 > 10^6^ bp, scaffold NG50 > 10^7^ bp, most of the genome separated into haplotypes (P2), Phred-scaled base accuracy > Q40, and 99.5% of the assembly assigned to chromosomes. The contig NG50 of the tufted duck genome assembly was 17.8 Mb. The curation (as in Howe et al. [38]) resulted in 34 removals of misjoins, 34 joins previously missed, and 4 removals of false duplications. This reduced the primary assembly length by 0.8% and increased the scaffold NG50 by 18.7% to 85.9 Mb whilst decreasing the scaffold number from 123 to 105. The total sequence length of the assembly after curation was 1.127 Gb, which is close to the expected size of duck genomes (1.25–1.34 Gb) [40]. During curation, telomeres were not detected, and a sweep for centromeres with sequences from [41] revealed no results. Chromosomal-scale units were identified and named by size. Of the expected 39 chromosome pairs according to the karyotype [42], 34 autosomes plus Z and W could be identified, with 99.6% of the sequence assigned to them (Table 1, Fig. 1). The pseudoautosomal region in W/Z (∼2 Mb) collapsed into a single copy on the Z chromosome (Supplementary Fig. S1). Detailed assembly statistics by chromosome can be found Supplementary Table S1.

**Table 1:** Assembly statistics of the tufted duck genome

Statistic	Value
Genome coverage (×)	64.03
Total sequence length (bp)	1,127,004,725
Ungapped sequence length (bp)	1,117,587,328
No. of scaffolds	105
Scaffolds assigned to chromosomes	36 + 1 mitochondrion
Unplaced scaffolds	68
Contig NG50 (bp)	17,816,505
Scaffold NG50 (bp)	85,905,639
Base-call error in 10 kb	<1 nucleotide

The raw data have been deposited in the GenomeArk repository [43]. The curated primary assembly has been deposited in NCBI under accession No. GCF_009819795.1 [44] and can be browsed in the Genome Data Viewer [45]. A comparison of assembly metrics before and after curation can be found in Supplementary Table S1.

### Analysis of repetitive sequences

Repetitive element content and composition in the tufted duck genome assembly was identified and compared with that of the mallard genome (ZJU1.0). Overall, 13% and 15% of the tufted duck and mallard genomes, respectively, are made up by repeats. The most abundant repeat classes are long interspersed nuclear elements followed by long terminal repeats, short interspersed nuclear elements, and DNA repeats. The genomic repeat composition, in general, is similar between tufted duck and mallard, although the mallard genome has slightly higher repeat content. The only major difference we observed was in repeats that were not classified by RepeatModeler into any known repeat categories. These are termed “Unknown,” and the mallard genome seems to contain 4.4 times as many Unknown repeats as the tufted duck genome (Supplementary Fig. S2). Nevertheless, when compared at chromosome level (Supplementary Fig. S3), repeat content is similar between the orthologous pairs, including autosomes and sex chromosomes, and the major difference is only observed in unplaced scaffolds. Forty percent of the mallard unplaced scaffolds are made up by Unknown repeats, while it is only 6% in the tufted duck.

Thirteen telomeric and 3 potential centromeric repeat regions were identified in the tufted duck genome (Supplementary Fig. S4).

### Gene/transcript model reconstruction with Illumina RNA-Seq and PacBio Iso-Seq reads

With the Illumina RNA-Seq, a mean (SD) of 97.65% (0.51%) of the reads were retained after adapter and quality trimming. For the PacBio Iso-Seq, a mean (SD) of 80.57% (3.84%) full-length non-chimeric (FLNC) eads were retained after error correction (Table 2).

**Table 2:** Illumina RNA-Seq reads before and after trimming, PacBio Iso-Seq reads before and after error correction, ZMW yield, and the number of FLNC reads

Platform	Parameter	Brain	Ileum	Lung	Ovary	Spleen	Testis
Illumina	No. raw reads (PE)	71,648,303	72,410,462	63,191,981	72,442,392	68,315,029	53,747,262
	No. trimmed (paired)	70,025,835	70,902,250	61,059,701	70,892,177	66,813,691	52,652,923
PacBio	No. subreads	9,249,099	12,363,369	19,206,097	1,279,561	8,401,115	12,616,953
	No. CCS	158,698	415,314	529,108	68,112	167,077	288,984
	ZMW yield (%)	15.87	41.53	26.46	3.41	16.71	28.90
	Mean No. of passes	58.3	29.8	36.3	18.8	50.3	43.7
	No. FLNC	133,684	343,634	432,817	49,887	134,124	234,423

Two ZMW were used for lung and ovary. CCS: circular consensus sequencing; FLNC: full-length, non-chimeric; PE: paired end; ZMW: zero-mode waveguide.

HISAT2 could map a mean (SD) of 93.21% (0.64%) Illumina RNA-Seq short reads and Minimap2 could map 97.39% (1.34%) PacBio Iso-Seq long reads to the reference genome. The mean (SD) read length of the long-read data was 1,214.0 (262.0) nt, an order of magnitude longer than that of the short reads (131.5 [0.7] nt). However, the mean number of reads was almost 600-fold higher with short-read data (129,411,992 [11,989,532]) compared to long-read data (217,293 [143,219]), implying a 60 times higher sequencing depth with the short reads. Not surprisingly, StringTie2 (in the short-read pipeline) assembled more transcript models and inferred more genes and exons than the long-read pipeline. This was true for all tissues except in the lung; more transcript models were inferred in the long-read pipeline. Lung RNA (cDNA) was sequenced on 2 Zero-mode waveguides (ZMW) and produced the highest numbers of subreads and FLNC after processing. The highest number of genes in each pipeline was predicted for ovary (Illumina) and brain (PacBio). The highest number of transcript models was predicted for ovary (Illumina) and lung (PacBio). The same pattern applies to predicted exons (Table 3).

**Table 3:** Transcript model reconstruction per tissue and pipeline

Parameter	Platform	Brain	Ileum	Lung	Ovary	Spleen	Testis
Mapped (%)	Illumina	92.92	92.98	93.42	94.33	92.44	93.19
	PacBio	96.66	98.13	97.92	99.40	95.67	96.57
No. genes	Illumina	22,348	20,838	20,692	32,046	21,608	29,225
	PacBio	15,776	10,813	12,912	8,862	6,773	11,746
No. transcripts	Illumina	44,808	40,968	43,877	77,997	42,000	57,758
	PacBio	37,601	35,284	46,587	19,513	14,030	28,852
No. exons	Illumina	422,741	395,719	412,225	569,566	383,679	483,444
	PacBio	138,038	217,391	243,566	119,080	71,211	160,380
Exons per gene (mean)	Illumina	18.9	19.0	19.9	17.8	17.8	16.5
	PacBio	8.7	20.1	18.9	13.4	10.5	13.7
Transcripts per gene (mean)	Illumina	2.0	2.0	2.1	2.4	1.9	2.0
	PacBio	2.4	3.3	3.6	2.2	2.1	2.5

**Figure 2:**
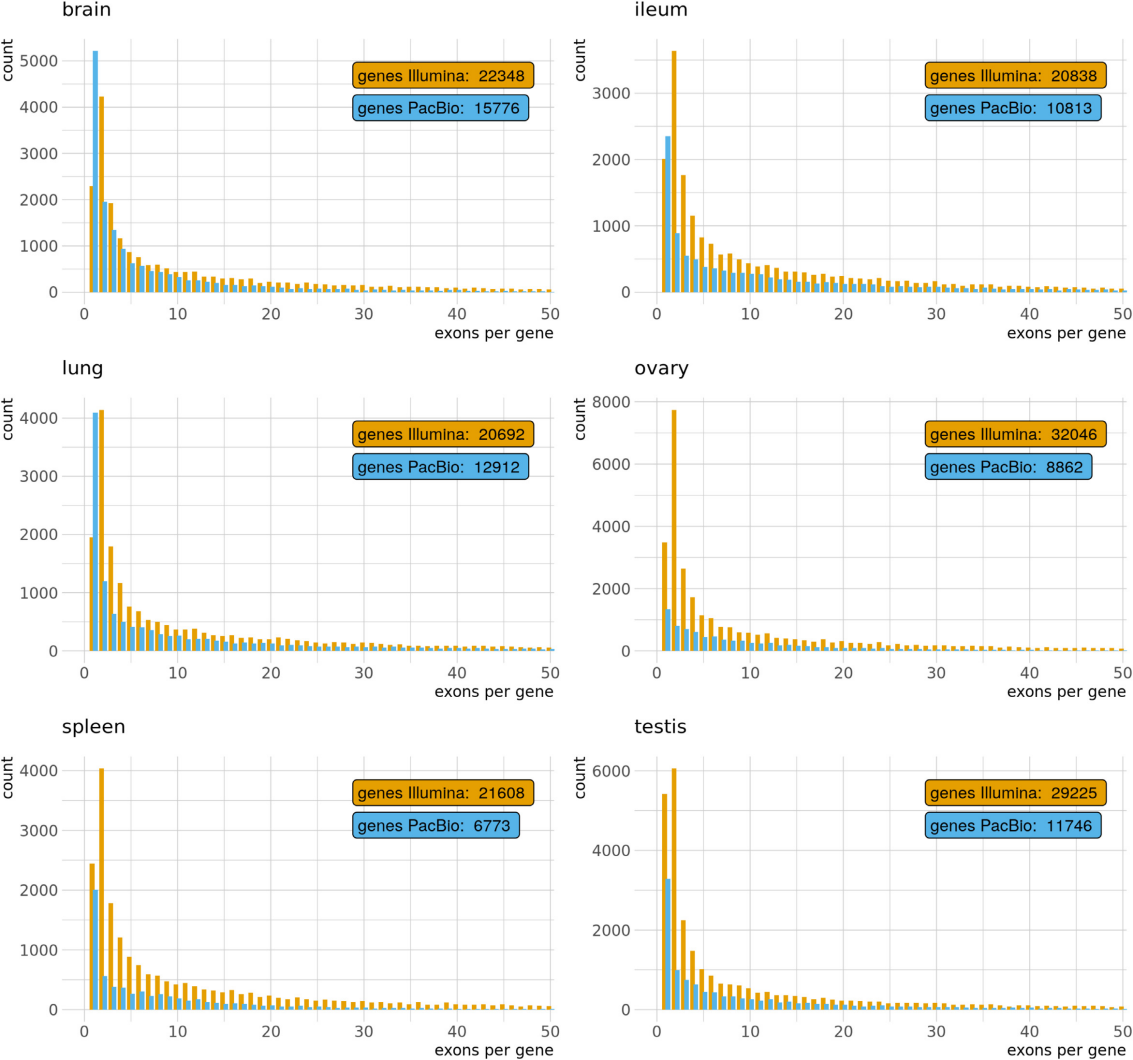
Distribution of single- and multi-exon genes per tissue and pipeline. Only the first 50 groups are shown.

In the Illumina short-read RNA-Seq transcript model reconstruction, more exons per gene were recovered on average than in the long-read pipeline (Table 3). The distribution of exons per gene in both pipelines generally followed the same pattern, with a decreasing number of genes as the exons per gene increased. However, there is a remarkable difference in the short-read data, which recovered more 2-exon genes than single-exon genes for all tissues (Fig. 2). This pattern is even more pronounced in the exons per transcript analysis (Supplementary Fig. S5).

**Figure 3:**
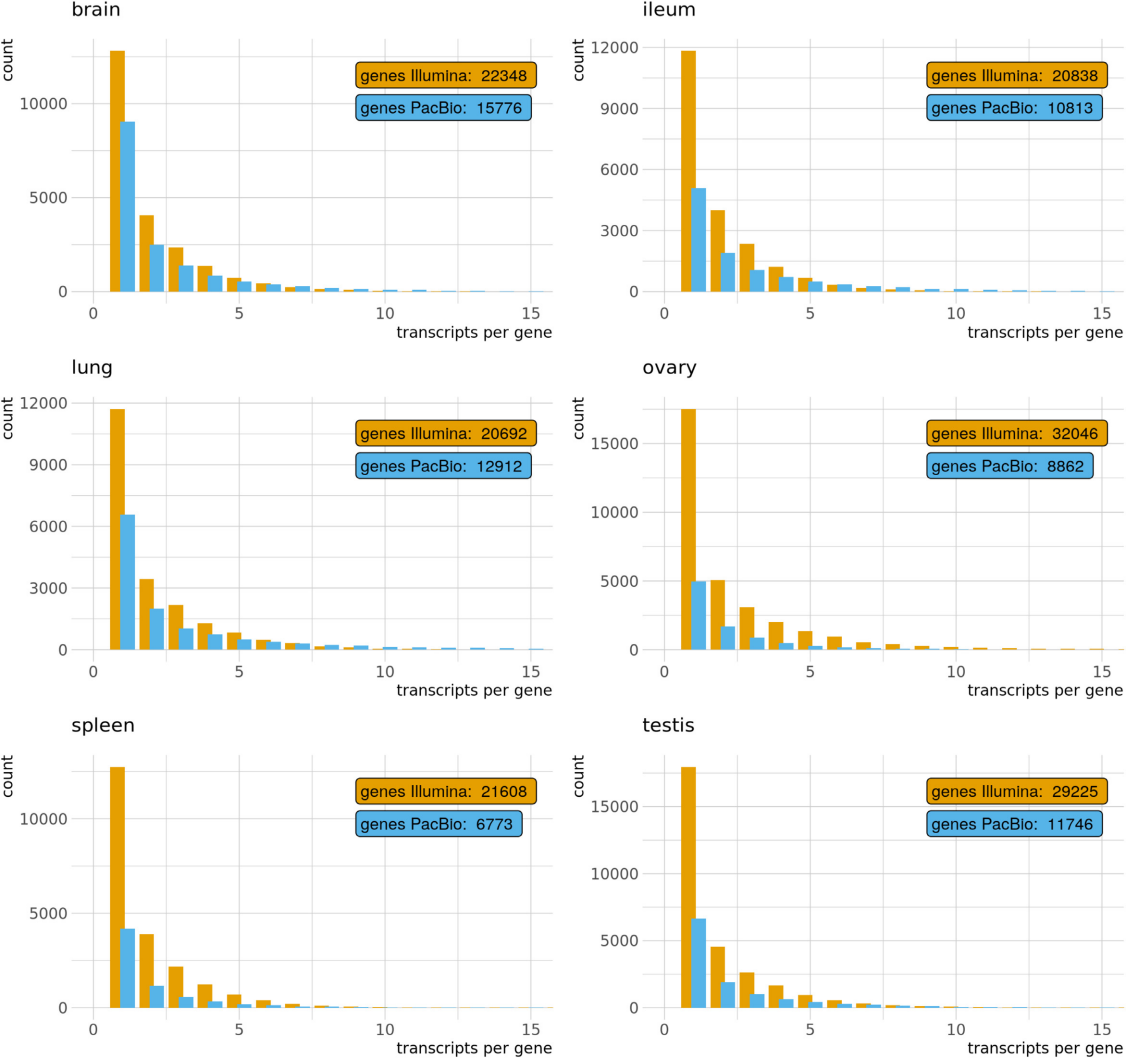
Distribution of single- and multi-transcript genes per tissue and pipeline. Only the first 15 groups are shown.

In the PacBio long-read Iso-Seq transcript model reconstruction, more transcripts per gene were recovered on average than in the short-read pipeline (Table 3). The distribution of transcripts per gene followed the same pattern, with a decreasing number of genes as the transcripts per gene increased. However, these numbers had a slower decay in the long-read data, indicating a better recovery of multi-transcript genes (Fig. 3).

### Functional annotation of merged transcripts

After merging all 12 transcript model reconstructions (6 tissues with short reads and long reads), 345,870 transcripts with 2,381,662 exons were predicted in 49,746 genes (Table 4). Of these, 178,198 transcripts (or 16,758 genes) were predicted by CPC2 to be protein-coding. UniProt hits were found for 208,274 transcripts (or 17,911 protein-coding genes). Of these, 4,766 genes had no long-read support and 432 genes no short-read support in the data. The number of protein-coding genes predicted by CPC2 and number of hits in UniProt overlapped for 15,103 genes (Supplementary Fig. S6). Conservative filtering of the annotation (ignoring features flagged as nonsense-mediated decay and only keeping full-length hits in UniRef50 that matched with ≥90%) resulted in 111,934 transcripts and 14,099 protein-coding genes. This result implies a mean of 7.9 isoforms per gene (Table 4). Predicted and annotated genes by chromosome can be found in Supplementary Table S2.

**Table 4:** Functional annotation categorized by different matches

Parameter	Genes	Transcripts	Isoforms
Total No. of entries	49,746	345,870	7.0
UniRef50 total hits	17,911	208,274	11.6
UniRef50 match			
Full	13,024	99,737	7.7
90%	4,937	12,197	2.5
50%	3,208	6,540	2.0
<50%	2,474	4,447	1.8
≥50%	14,731	118,474	8.0
≥90%	14,099	111,934	7.9
No hit (but full-length)	27,787	78,860	

Note: UniRef50 total hits also includes 5′ degraded records, whereas the match classes only include full-length records.

Of 78,860 full-length transcripts with no UniRef50 hit, 62,147 were flagged as potentially protein-coding (and the remainder as nonsense-mediated decay), and 26,489 as single-exonic while the remainder as composed of ≥2 exons.

### RIG-I/DDX58 is intact and expressed in tufted duck

In the mallard genome [46,47], *RIG-I/DDX58* is annotated on chromosome Z (NC_051804.1), position 69,037,671–69,061,400 (23,730 nt), and consists of 18 exons. Searching the protein sequence in the tufted duck genome assembly produced 1 significant alignment with the predicted gene *XM_032205362.1*, also on chromosome Z (max score: 1,882, total score: 1,882, query cover: 100%, E-value: 0.0, percent identity: 97.00%; see alignment in Supplementary Listing 1).

Searching the predicted open reading frames (ORFs) from the functional annotation in the 2 mallard *RIG-I/DDX58* isoforms revealed 9 matches after conservative filtering (≥90% match; Table 5). Gene *G24916* on chromosome 6 (NC_045564.1) matched with ORFs of 6 transcripts while G24916.1 contained the same translated amino acid sequence as G24916.2 and G24916.4. Gene *G46857* on chromosome Z (NC_045593.1) matched with ORFs of 3 transcripts while *G46857.2* contained the same translated amino acid sequence as G46857.3. ORFs G24916.7/8 and G46857.2/3/4 were flagged with “5prime_degrade," which means that the transcript might be incomplete on the 5′ end. Both genes were predicted in the short-read and long-read pipeline. A detailed list of reconstructed transcript models by pipeline and tissue for these 2 genes can be found in Supplementary Table S3.

**Table 5:** Blastp results (≥90% match) of predicted ORFs from the functional annotation searched in 2 mallard *RIG-I/DDX58* isoforms NP_001297309.1 (933 aa) and XP_038025643.1 (988 aa)

Mallard	Tufted duck							
Isoform	Chromosome	ORF	Start/End	nt	Frame	Strand	Exons	aa
NP_001297309.1	NC_045564.1 (6)	G24916.1	21,885,153/21,914,958	29,805	F2	+	17	1,003
NP_001297309.1	NC_045564.1 (6)	G24916.2	21,885,153/21,914,958	29,805	F1	+	16	1,003
NP_001297309.1	NC_045564.1 (6)	G24916.3	21,887,313/21,915,355	28,042	F1	+	17	994
NP_001297309.1	NC_045564.1 (6)	G24916.4	21,887,313/21,914,044	26,731	F1	+	16	1,003
XP_038025643.1	NC_045564.1 (6)	G24916.7*	21,887,508/21,915,353	27,845	F1	+	16	1,044
XP_038025643.1	NC_045564.1 (6)	G24916.8*	21,887,508/21,915,503	27,995	F1	+	16	1,040
NP_001297309.1	NC_045593.1 (Z)	G46857.2*	69,123,499/69,145,704	22,205	F3	+	18	948
NP_001297309.1	NC_045593.1 (Z)	G46857.3*	69,123,499/69,147,273	23,774	F3	+	18	948
NP_001297309.1	NC_045593.1 (Z)	G46857.4*	69,123,529/69,145,281	21,752	F3	+	18	938

ORFs marked with an asterisk were flagged with “5prime_degrade," which means that the start codon was not found in the TAMA ORF/NMD prediction pipeline. aa: amino acid.

All *G24916* sequences relate to the *IFIH1* gene (encodes MDA5, a RIG-I-like receptor) while *G46857* sequences relate to the *RIG-I/DDX58* gene. An alignment of the mallard and tufted duck *RIG-I/DDX58* amino acid sequences revealed 15 variants (14 substitutions, 1 insertion), which were predicted to have no effect on the biological function of the protein (Supplementary Tables S4 and S5). The nucleotide sequences 1 kb upstream of the *RIG-I/DDX58* gene contained 4 identical transcription factor binding sites (Nkx2-5, NF-kappB p65, c-Rel, NF-kappaB) in each species (Supplementary Table S6).

### Tissue-specific expression and intersections

**Figure 4:**
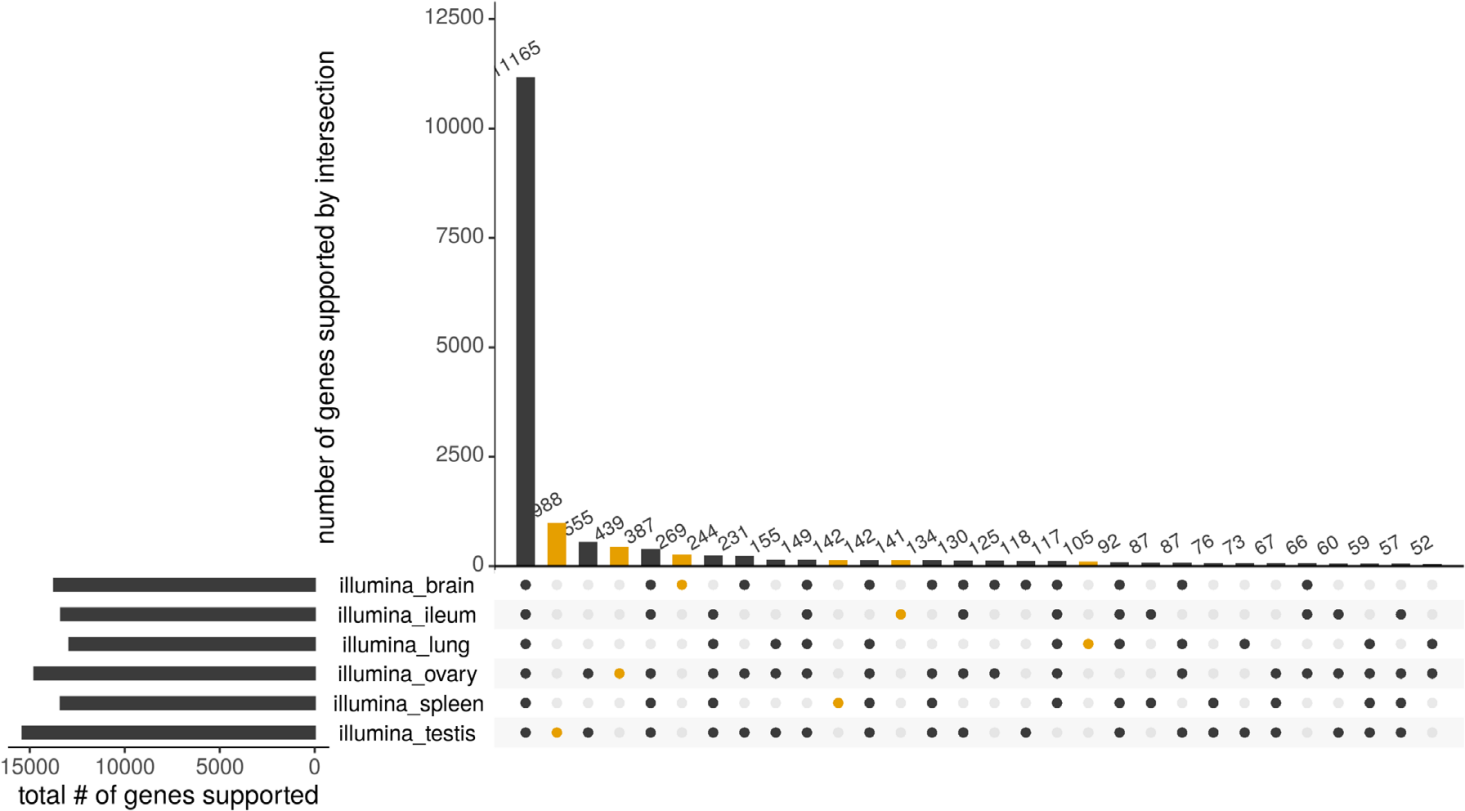
In the short-read data set, the highest total number of supported genes was found in testis (left panel, bottom), followed by ovary, brain, spleen, ileum, and lung. All 6 tissues intersected in 11,165 genes (main panel, left). The highest number of exclusively supported genes was also found in testis (988), and followed the same order as the total number of genes (main panel, yellow).

**Figure 5:**
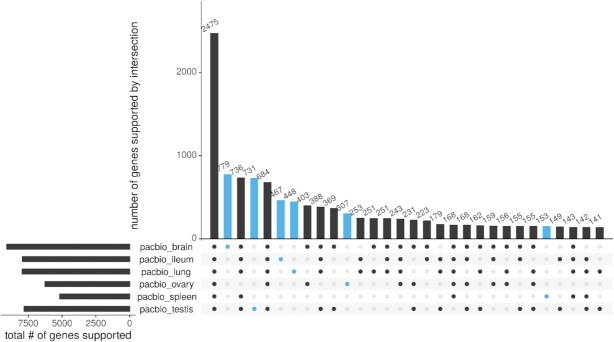
In the long-read data set, the highest total number of supported genes was found in brain (left panel, top), followed by lung, ileum, testis, ovary, and spleen. All 6 tissues intersected in 2,475 genes (main panel, left). The highest number of exclusively supported genes was found in brain (779), followed by testis, ileum, lung, ovary, and spleen (main panel, blue).

Of the 17,911 genes in UniRef50 (total hits), 4,766 were exclusively supported by short reads and 432 by long reads. In the short-read pipeline, 11,165 genes intersected across all tissues. The highest number of exclusively expressed genes was in testis (988), followed by ovary, brain, spleen, ileum, and lung (Fig. 4). In the long-read pipeline, 2,475 genes intersected across all tissues. The highest number of exclusively expressed genes was in brain (779), followed by testis, ileum, lung, ovary, and spleen (Fig. 5). Overlap of expressed genes from each pipeline can be found in Supplementary Fig. S7.

### Small RNA analyses

For the TruSeq small RNA sequencing data, a mean (SD) of 95.81% (3.51%) of the reads were retained after adapter and quality trimming (Table 6). Overall, star could map a mean (SD) of 93.91% (5.28%) of these reads to the reference genome, which divides into 67.99% (13.75%) uniquely mapped reads and 25.92% (12.08%) multi-mapped reads (≤10 loci). Cufflinks predicted the highest number of genes in the spleen (33,133) followed by testis (31,205). The remaining tissues had much lower numbers of genes, ranging from 8,441 (ileum) to 13,606 (brain). The same pattern applies to the number of predicted transcripts and exons (Table 6).

**Table 6:** Small RNA read processing and assembly statistics

Statistic	Brain	Ileum	Lung	Ovary	Spleen	Testis
No. raw reads (PE)	78,078,195	58,021,264	70,381,224	79,425,103	65,767,638	67,436,837
No. trimmed reads (PE)	73,753,404	57,021,189	69,326,112	77,333,115	58,716,681	65,395,835
Mapped uniquely (%)	51.82	88.04	72.04	71.01	72.43	52.59
Mapped multiply (%)	44.90	9.48	24.42	26.59	18.18	31.97
No. genes	13,606	8,441	11,899	9,903	33,133	31,205
No. transcripts	13,685	8,520	11,995	9,954	33,761	31,342
No. exons	17,276	12,650	15,397	11,588	54,504	35,026

PE: paired end.

**Figure 6:**
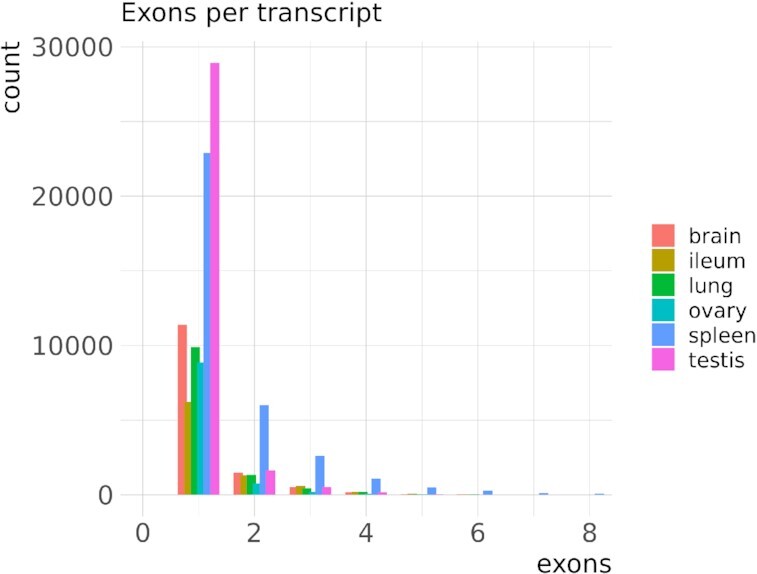
Distribution of single-exon and multi-exon small RNA transcripts for each tissue.

Each transcript was composed of a mean (SD) of 1.3 (0.2) exons. The distribution of single-exon and multi-exon transcripts shows a clear trend towards single-exon transcripts and a quickly diminishing number of multi-exon transcripts. However, this was less pronounced in spleen but even more so in testis (Fig. 6). The generally decaying length distribution of transcripts shows 2 clear peaks at 20 and 50 bp except for testis with the first peak at 30 bp. Except for spleen and testis data, there are very few transcripts >200 bp (Supplementary Fig. S8).

Scanning the genome *(in silico)* for Rfam’s covariance models of RNAs resulted in 1,234 hits. The same scan on the assembled small RNA transcripts *(in vitro)* revealed a mean (SD) of 346.5 (26.9) hits across all tissues. After removing lower-scoring overlaps and hits with E-value >5.0E−4 from the cmscan result, 1,076 distinct features were predicted in the tufted duck genome. In the tissue’s small RNA assemblies, a mean of 327.5 (26.5) features were annotated, with the same filtering (Table 7). A mean (SD) 93.25% (1.09%) of the *in vitro* annotated features were predicted by the *in silico* scan. Furthermore, a mean (SD) of 22.2 (2.3) features were annotated that were not predicted *in silico* (Table 7).

**Table 7:** Results of cmscan on assembled small RNA transcripts after filtering

Parameter	Brain	Ileum	Lung	Ovary	Spleen	Testis
*In vitro*	328	294	317	312	345	369
Intersection	310	274	295	293	315	345
Additional	18	20	22	19	30	24

Intersection refers to small RNAs predicted by the *in silico* genome scan. Additional refers to annotated small RNAs that were not detected by cmscan in the reference genome.

After further filtering for unique RNA families (Rfam accession numbers/covariance models), 306 distinct RNA families were predicted in the genome, with 246 annotated in all tissues (pooled). The number of predicted and annotated covariance models overlapped for 237 RNA families, while 69 were only identified in the genome scan and 9 were only identified in the pooled tissue annotations.

## Discussion

In this study, we present the first chromosome-level reference genome assembly of the tufted duck. The genome contiguity is on par with other reference bird genome assemblies that used long reads such as mallard [46,47], chicken [48], and recent VGP-pipeline–generated zebra finch [49]. The assembly’s contig NG50 of 17.8 Mb is comparable to chicken (17.7 Mb) but considerably higher than in mallard (5.7 Mb) and zebra finch (4.4 Mb). The assembly’s scaffold NG50 of 85.9 Mb is higher than in mallard (76.3 Mb) and zebra finch (70.9 Mb) and considerably higher than in chicken (20.8 Mb). All our mapping results from the Illumina short-read and PacBio long-read RNA pipelines confirm full adherence to the VGP 6.7.P2.Q40.C99 standard, which also implies transcript mappability >80% [24].

The higher numbers of recovered genes, transcripts, and exons in the short-read transcript model reconstruction can be mainly explained by the higher sequencing depth and further reinforced by the different RNA preparation protocol. With Illumina, virtually all trimmed, paired-end reads were kept for mapping to the genome while with PacBio, only 5′ cap-selected and FLNC reads were kept. However, the transcript model reconstruction in the long-read pipeline often almost matched or even exceeded (lung) the mean number of transcripts in the short-read pipeline. Furthermore, the long-read pipeline recovered more transcripts per gene (isoforms) on average. Taken together, this is remarkable considering the 60-fold higher sequencing depth in the short-read pipeline. It also corroborates the strength of PacBio Iso-Seq, which seems to better reflect the complexity of the transcriptome, considering that transcripts did not need to be assembled but were sequenced full-length. However, this result is not reflected in the functional annotation and, together with the 27,787 putatively intact genes without a hit in the UniRef50 database, may indicate potentially undescribed genes.

In the short-read transcript model reconstruction, more 2-exon genes than single-exon genes were predicted for all tissues, and it seems as if some transcripts could not be assembled entirely or StringTie2 tried to “avoid” single-exon genes. Transcript model reconstruction in StringTie2 is based on the concept of extending short reads to create so-called super-reads [50], which seems appropriate for whole-genome assemblies. In transcriptomics, however, multiple splice variants are possible, and with the super-read concept in mind, it may thus be possible that StringTie2 discards a single-exon transcript model in favour of an alternative multi-exon splice variant containing the same exon. This, in turn, might have a substantial impact on the functional annotation based on transcript models solely assembled with short reads. Real single-exon transcripts might be missed, or even worse, false-positive multi-exon transcript models might be reconstructed. We merged transcript models of both pipelines with higher priority on transcript end sites for the long-read–inferred transcript models to mitigate this effect.

While both the number and quality of published vertebrate genome assemblies are increasing, hardly any are complemented by a transcriptome of multiple tissues from the same individual [26,51]. Automated annotations (e.g., the NCBI Eukaryotic Genome Annotation Pipeline [52]) provide reasonable *in silico* predictions of coding potential; however, RNA (cDNA) sequencing adds evidence for expressed genes. Based on the inferred transcripts in this study, a total of 14,099 protein-coding genes could be identified in the UniRef50 database after conservative filtering (≥90% match), which is comparable to NCBI’s *in silico* prediction of 15,578 protein-coding genes [53]. The number of identified protein-coding genes in tufted duck is also in line with the prediction in other bird species such as mallard (16,836 [54]), chicken (17,477 [55]), or zebra finch (16,197 [56]). CPC2 predicted 84.2% of the potentially protein-coding genes found in UniRef50, which would serve as a conservative estimate of the protein-coding potential by just looking at the reference genome. However, beyond the 17,911 genes annotated by UniRef50, the annotation contains an additional 27,787 genes with protein-coding potential according to the TAMA ORF/NMD prediction pipeline, with these being potential candidates for further analyses. Gene and transcript identification in non-model species relies on annotations of preferably closely related model organisms. However, protein-coding genes are mainly described by a single transcript and predominantly built on short-read and comparative data [26].

We could confirm that the gene *RIG-I/DDX58* is intact and expressed in the tufted duck, and almost identical and at the same position as in mallard. The differences in amino acid sequence are tolerated, and transcription factor binding sites are identical with those in mallard. Taken together, there are no obvious differences in the *RIG-I* gene that can account for the difference in susceptibility to influenza seen in each species. This is remarkable considering that tufted ducks are highly susceptible to AIV and indicates that the host response is complex and depends on more than an intact and expressed *RIG-I/DDX58* gene [36,39].

Besides a high-quality transcriptome for the tufted duck, this study also provides a tissue-specific expression atlas. In the short-read pipeline, there is a large decrease in numbers of genes expressed in all tissues to genes exclusively expressed in a single tissue or a few tissues. This distribution is much more balanced in the long-read pipeline and may indicate that the coverage in PacBio was too low to fully recover all genes in all tissues.

The number of 306 Rfam small RNA families predicted (with 246 annotated) for the tufted duck in this study is comparable to 352 families predicted in chicken (*Gallus gallus*, version 5) in Rfam 14.4 [57]. The peaks of transcript length at 20 and 50 bp are in the area of microRNA (miRNA), which are usually 18–23 nt [58–60], and pre-miRNA, which are in the range of 55–70 nt. The substantial variance across tissues in our data (many more genes, transcripts, and exons in spleen and testis than in the other tissues) might be explained by Illumina sequencing bias introduced at the adapter-ligation step of cDNA library constructions [61,62]. Furthermore, according to Illumina’s TruSeq Small RNA library preparation protocol, small RNA populations can vary significantly between different tissue types and species, and types and coverage vary depending on which bands are selected during gel excision. This is additional support of a strategy to sequence multiple tissues to obtain a fuller picture of small RNA expression in an organism. Gene duplications might explain the significant difference between unique mappings and multiple mappings of small RNA to the genome across tissues. The *in silico* scan of small RNA in the genome could predict almost all small RNAs in the assembled transcripts. More importantly, however, 22.2 additional small RNAs were discovered on average in the *in vitro* scan that would otherwise have been unnoticed. Small (non-coding) RNAs play an essential role in gene regulation, translation, and chromosome structure [63,64] and are often associated with diseases [65,66]. This vital fraction of the genome is rarely validated *in vitro* in the genome and transcriptome annotation literature. However, small RNA studies have been continuously increasing over the past 20 years from 1,966 publications in 1999 to 8,034 in 2019 (searching for “small RNA” on Web of Science [67]). Relying on *in silico* prediction of small RNA alone can lead to misinterpretation of pathways and gene regulation, and sequencing small RNA of non-model organisms is, therefore, an advancement for genome annotation [68]. We strongly encourage *in vitro* sequencing of small RNA in *de novo* genome and transcriptome studies, or otherwise, the scientific community will miss much detail that will be important to decipher relevant differences in the biology between species.

## Potential Implications

This study presents the first high-quality reference genome assembly of the non-model tufted duck species. It is complemented by coding and small non-coding RNA transcriptome annotation from 6 different tissues. The genome assembly contributes to the VGP’s ongoing mission to generate near error-free and complete genome assemblies of all extant vertebrate species. By utilizing, comparing, and combining the strengths of low error rates and high sequencing depth in Illumina RNA sequencing, and the full-length transcript sequencing in PacBio’s Iso-Seq, this annotation culminates in a merged transcriptome with functional annotation and an expression atlas. Evidence from small RNA of the same tissues sequenced using the Illumina platform revealed small RNAs that would have otherwise remained undetected. Our findings from a comparison between short-read and long-read reference transcriptomics contribute to a deeper understanding of these competing options. In this study, both technologies complemented each other. While short-read data were sufficient to annotate protein-coding genes, long-read data recovered more transcripts per gene and potentially further protein-coding genes that could not be annotated. With the ongoing improvement of base call quality in long-read sequencing, short-read transcriptome sequencing might become expendable, and we recommend reconstructing transcriptomes using long-read sequencing with high coverage. Together, the genome and transcriptome annotation of the tufted duck is an excellent resource for public omics databases and a foundation for downstream studies, e.g., regarding disease response. The data set’s high quality for a non-model species allows for a much finer resolution of genetic differences and commonalities in closely related species, which is crucial while studying the reservoirs of zoonotic pathogens.

## Methods

### Sampling and dissection of tissues

Captive-bred, wild tufted ducks were kept at the animal breeding facility (Swedish National Veterinary Institute, Uppsala, Sweden). The ducks were obtained from Snavelhof breeding farm, Veeningen, the Netherlands, in May 2017. Tissue samples were obtained from 5 females and 5 males (12 months old) after euthanasia with an injection of 1 mL of pentobarbital (100 mg) in the wing vein. The following tissues were collected from the birds: brain, ileum, spleen, lung, and gonads (ovary or testis). Tissues were immediately snap-frozen in liquid nitrogen and stored at −80°C until shipment on dry ice to the Roslin Institute, Edinburgh, UK. All animal experiments were carried out in strict accordance with a protocol legally approved by the regional board of the Uppsala animal ethics committee, Sweden (permission No. 5.8.18-07998/2017). The animal experiments were conducted in biosafety level 2 animal facilities at the Swedish National Veterinary Institute.

### Genomic DNA: library preparation, sequencing, and assembly

To obtain both sex chromosomes, DNA was extracted from lung tissue of a female tufted duck. Library preparation and sequencing was conducted as in [24], using 4 types of sequencing data and the VGP assembly pipeline 1.6 (all details given in Rhie et al. [24] and pipeline available on [69]). The sequence data consisted of PacBio chromium linked reads (CLRs) (64.03× coverage), 10X Genomics CLRs (110.83× coverage), Bionano Genomics optical maps created by direct labelling and staining (DLS; 371.51× coverage), and chromatin conformation capture coupled with high-throughput sequencing (Hi-C; 92.27× coverage) (Arima Genomics, San Diego, CA, USA). In brief, PacBio reads were input to the diploid-aware long-read assembler falcon and its haplotype-resolving tool FALCON-Unzip [70]. The resulting primary contigs were input to the Purge-Dups pipeline [71] to identify and remove remaining haplotigs in the primary set. In the next step, primary-purged contigs were subject to 2 rounds of scaffolding using the 10X long molecule linked reads. Further, pre-assembled DLS Bionano cmaps were applied for further scaffolding and ordering using the Solve pipeline (Bionano Genomics, San Diego, CA, USA). The resulting scaffolds were then further scaffolded into chromosome-scale scaffolds using Hi-C data and the Salsa2 pipeline [72]. Finally, the primary assembly plus the Falcon-phased haplotigs were concatenated for 3 rounds of base call polishing: first with PacBio reads and Arrow software [73] and subsequently 2 rounds of polishing with 10X linked reads and FreeBayes software (FreeBayes, RRID:SCR_010761) [74]. The genome was decontaminated and manually curated as described in Howe et al. [38].

### Tissue preparation, DNA and RNA extraction

For disruption and homogenization of tissues, snap-frozen samples were ground to a fine powder under liquid nitrogen using a mortar and pestle. Samples were transferred to 1.5 mL frozen tubes and kept on dry ice until further processing. Total RNA was obtained following a standard TRIzol protocol with DNase treatment and column purification. Small RNA was prepared according to the miRNeasy kit protocol 217004 (Qiagen, Venlo, Netherlands). Integrity and quality of the RNA were confirmed by electrophoresis on an Agilent 2200 Tapestation using appropriate screen tapes. The concentration was determined using the Nanodrop ND-1000 (Thermo Fisher Scientific, Waltham, MA, USA) (Supplementary Tables S7 and S8). For DNA extraction and sequencing, powdered lung tissue was sent to the Vertebrate Genomes Lab (VGL) at Rockefeller University, New York, NY, USA.

### Illumina cDNA library preparation and sequencing

RNA was sent to Edinburgh Genomics, Edinburgh, UK, for library preparation and sequencing on an Illumina HiSeq 4000 platform (Illumina HiSeq 3000/HiSeq 4000 System, RRID:SCR_016386) [75] with 2 × 150 bp paired-end reads using the TruSeq library preparation protocol (stranded). Median (SD) insert size was 137–148 (67–81 bp), yielding ≥290 M + 290 M reads per sample. Small RNA was also sent to Edinburgh Genomics for TruSeq small RNA library preparation and sequencing using a NovaSeq 6000 platform (Illumina NovaSeq 6000 Sequencing System, RRID:SCR_016387) [76] with 2 × 50 bp paired-end reads. Median insert size was 141–144 bp, yielding ≥225 M + 225 M reads per sample.

### Genome analysis and comparison with the mallard genome assembly

Repeat content in the tufted duck genome assembly was defined using RepeatMasker v4.1.0 (RepeatMasker, RRID:SCR_012954) [77] with duck-specific repeat sequences from the combined Dfam v3.1 (Dfam, RRID:SCR_021168) [78] and RepBase v20170127 (Repbase, RRID:SCR_021169) [79] repeat libraries. RepeatMasker was run in “sensitive" mode (-s) using “*Aythya fuligula*” as the query species (-species ‘Aythya fuligula’). This was followed by a second round of repeat masking, which was carried out using a novel repeat sequence library obtained by RepeatModeler v2.0.1 (RepeatModeler, RRID:SCR_015027) [80]. To generate a comparative data set on the mallard genome (ZJU1.0 [46,47]) we used the same repeat-masking strategy.

Telomeric repeats were identified by searching for known vertebrate-specific repeat hexamers of “TTAGGG” and “CCCTAA,” while known Anseriformes-specific centromeric repeat sequences [81] were mapped with RepeatMasker.

Orthologous chromosome pairs were identified by searching for synteny between the tufted duck and mallard genomes. The 2 genomes were aligned with Minimap2 (Minimap2, RRID:SCR_018550) [82] using options “–secondary=no -asm 10" and the primary alignments were used as a proxy for synteny between the 2 genomes. Primary alignments between regions are shown in a circos plot [83] in Supplementary Fig. S9.

### PacBio cDNA library preparation and sequencing

Two micrograms of total RNA from each sample in 4 parallel reactions were converted to cDNA using the Teloprime full-length cDNA amplification kit (013, v1) according to the manufacturer’s instructions (Lexogen, Vienna, Austria). After end-point PCRs, all samples were tested for quality and quantity. The product size distribution was visualized using an Agilent 2200 Tapestation using D5000 screen tape. The library concentration was measured on a Qubit 3 (Thermo Fisher Scientific, Waltham, MA, USA) with high-sensitivity DNA reagents (Supplementary Table S9). Technical replicates were pooled and selected for PacBio Iso-Seq assays. The samples were sequenced at Edinburgh Genomics using Sequel (version 2.1) chemistry.

### Gene/transcript model reconstruction

Illumina raw RNA-Seq reads were quality checked and filtered using FastQC v0.11.8 (FastQC, RRID:SCR_014583) [84] and Trimmomatic v0.38 (Trimmomatic, RRID:SCR_011848) [85], respectively. Corrected reads were mapped to the genome using HISAT2 v2.2.0 (HISAT2, RRID:SCR_015530) [86,87] and transcript models assembled using StringTie2 v2.1.1 (StringTie, RRID:SCR_016323) [50]. The resulting transcript models file was converted with tama_format_gtf_to_bed12_stringtie.py. Hereinafter, all tools described as starting with “tama_” are part of the software suite Transcriptome Annotation by Modular Algorithms [88], except for tama_merge_report_parser.pl [89].

PacBio raw Iso-Seq reads were pre-processed using the IsoSeq3 pipeline to obtain full-length, non-chimeric reads (FLNC; first 3 steps in [90]; ccs v3.3.0, lima v1.8.0, refine v3.1.0). Afterwards, fasta sequences were extracted from bam files using Bamtools v2.5.1 (Bamtools, RRID:SCR_015987) [91] and poly-A tails were trimmed using tama_flnc_polya_cleanup.py (v20191022). These FLNC were mapped to the reference genome with the splice site–aware mapper Minimap2 v2.17-r974-dirty. Redundant transcript models were collapsed with the capped flag (-x capped) using tama_collapse.py when coverage was ≥95% (-c 95). Additionally, 5′ threshold and 3′ threshold (tolerance in bp for grouping reads) were set to 100 (-a 100 -z 100).

### Functional annotation

Transcript models from all 6 tissues inferred by the short-read and long-read pipelines were merged on the basis of similarity using tama_merge.py (options -a 100 -z 100 -d merge_dup) with different priorities for splice junctions and transcript end sites. Short-read inferred transcript models were given higher priority on splice junctions, whereas long-read inferred transcript models were given higher priority on transcript end sites. Nucleotide sequences based on coordinates in the merged transcriptome were extracted from the reference genome using Bedtools v2.29.0 (BEDTools, RRID:SCR_006646) [92]. The protein-coding potential was predicted with CPC2 v0.1 (Coding Potential Calculator, RRID:SCR_002764) [93] based on the transcripts’ sequence features. ORFs were predicted and translated into amino acid sequences using tama_orf_seeker.py. Putative protein-coding sequences were identified in the UniProt/UniRef50 database v2019_10 (UniRef, RRID:SCR_010646) [94] using Blastp v2.9.0 (BLASTP, RRID:SCR_001010) [95]. The results were filtered for top hits with tama_orf_blastp_parser.py, and a new annotation with coding sequence (CDS) regions was created using tama_cds_regions_bed_add.py.

### Identification of RIG-I/DDX58

The nucleotide sequence of the antiviral innate immune response receptor RIG-I/DDX58 in mallard (*Anas platyrhynchos*, version NP_001297309.1) was used to search the tufted duck genome assembly using default Tblastn (TBLASTN, RRID:SCR_011822) [96] settings on NCBI. The protein sequences of the mallard RIG-I/DDX58 were downloaded (2 isoforms) and the predicted tufted duck ORFs searched in these using Blastp (v2.10.0+). Matching ORFs were aligned with the mallard isoforms using Clustal (Clustal 2, RRID:SCR_017055) [97], and protein variation analysed using provean (PROVEAN, RRID:SCR_002182) [98] and sift (SIFT, RRID:SCR_012813) [99]. Transcription factor binding sites (TFBS) were identified 1 kb upstream of *RIG-I/DDX58* in each species and compared by searching the transfac database (TRANSFAC, RRID:SCR_005620) [100] with the software P-Match (Gene Regulation Programs, RRID:SCR_007787) [101].

### Tissue-specific expression analysis

In addition to merging transcript models, tama_merge.py also creates gene and transcript reports that trace the source (in this case: pipeline and tissue) of each gene and transcript, respectively. The gene report was parsed with tama_merge_report_parser.pl [89] and filtered for genes found in UniRef50: Each gene was assigned a binary TRUE or FALSE label depending on the support of each of the 12 possible sources (2 pipelines and 6 tissues). The result was loaded into UpSetR [102, 103] to visualize intersections of tissue-specific evidence for an expressed gene in each pipeline.

### Small RNA analyses

Illumina raw reads were quality checked with FastQC v0.11.8 and adapters removed with Cutadapt v2.10 (cutadapt, RRID:SCR_011841) [104]. Corrected reads were mapped to the reference genome using star v2.7.3a (STAR, RRID:SCR_004463) [105] and assembled with Cufflinks v2.2.1 (Cufflinks, RRID:SCR_014597) [106–109]. Nucleotide sequences were extracted from the reference genome at the assembled transcripts’ coordinates using Bedtools getfasta -split (v2.29.2), and transcript lengths extracted from the output of Samtools faidx (SAMTOOLS, RRID:SCR_002105) [110]. All plots were created using the package ggplot2 (ggplot2, RRID:SCR_014601) [111] in RStudio (RStudio, RRID:SCR_000432) [103,112].

The tool cmscan v1.1.3 from the software suite Infernal (Infernal, RRID:SCR_011809) [113] was used to predict structural RNAs in the reference genome *(in silico)* and to annotate assembled small RNA transcripts *(in vitro)* based on Rfam (Rfam, RRID:SCR_007891) [114,115] covariance models downloaded from [116].

The output of cmscan (tblout) was converted to gff3 annotation files using tblout2gff3.pl [89]. Shared intervals between *in silico* and *in vitro* annotations were identified with the intersect option of Bedtools v2.29.2 [92].

## Availability of Source Code and Requirements

Perl and R scripts used in this study are available on GitLab at https://gitlab.com/rcmueller/tufted_duck_annotation under MIT license.

## Data Availability

The data sets supporting the results of this article are available in NCBI and Figshare. The curated assembly of the tufted duck genome has been deposited in NCBI under accession No. GCF_009819795.1 [44]. The Illumina RNA-Seq and small RNA-Seq, and PacBio Iso-Seq raw reads have been deposited in NCBI under BioProject PRJNA561952 (SRA accession Nos. SRX9968577–SRX9968594). Supporting data have been deposited on Figshare: Illumina RNA-Seq transcript models [117], PacBio Iso-Seq transcript models [118], functional annotation [119], expression atlas [120], Illumina small RNA-Seq annotation [121], additional scripts [89]. All supporting data and materials are available in the *GigaScience* GigaDB database [122].

## Supplementary Material

giab081_GIGA-D-21-00072_Original_Submission

giab081_GIGA-D-21-00072_Revision_1

giab081_GIGA-D-21-00072_Revision_2

giab081_GIGA-D-21-00072_Revision_3

giab081_Response_to_Reviewer_Comments_Original_Submission

giab081_Response_to_Reviewer_Comments_Revision_1

giab081_Response_to_Reviewer_Comments_Revision_2

giab081_Reviewer_1_Report_Original_Submission Qi Zhou -- 4/7/2021 Reviewed

giab081_Reviewer_1_Report_Revision_1 Qi Zhou -- 8/6/2021 Reviewed

giab081_Reviewer_2_Report_Original_Submission Joshua PeÃ±alba -- 4/23/2021 Reviewed

giab081_Supplemental_Files

## Additional Files

**Supplementary Figure S1**. Hi-C contact map of *Aythya fuligula* visualised in HiGlass.

**Supplementary Figure S2**. Comparison of genomic repeat content between tufted duck and the mallard.

**Supplementary Figure S3**. Comparison of repeat and unique sequence content in orthologous chromosome pairs between tufted duck and mallard.

**Supplementary Figure S4**. Identified telomeric and centromeric repeats in the tufted duck genome assembly.

**Supplementary Figure S5**. Distribution of single- and multi-exon transcripts per tissue and pipeline.

**Supplementary Figure S6**. Protein-coding potential calculated by CPC2.

**Supplementary Figure S7**. Pipeline-tissue-specific expression of genes found in UniProt.

**Supplementary Figure S8**. Length distribution of small RNA transcripts.

**Supplementary Figure S9**. Orthologous chromosome pairs of tufted duck and mallard.

**Supplementary Table S1**. Assembly metrics before and after curation.

**Supplementary Table S2**. Assembly and annotation statistics.

**Supplementary Table S3**. Gene prediction and transcript model reconstruction.

**Supplementary Table S4**. Protein variation effect analysis.

**Supplementary Table S5**. SIFT amino acid substitution analysis.

**Supplementary Table S6**. Transcript factor binding sites analysis.

**Supplementary Table S7**. RNA extraction details.

**Supplementary Table S8**. Small RNA extraction details.

**Supplementary Table S9**. cDNA library details.

**Supplementary Listing 1**. Full alignment of antiviral immune response receptor (Aythya fuligula and Anas platyrhynchos).

## Abbreviations

AIV: avian influenza A virus; BLAST: Basic Local Alignment Search Tool; bp: base pairs; cDNA: complementary DNA; CLR: continuous long reads; DLS: direct label and stain; FLNC: full-length, non-chimeric reads; Gb: gigabase pairs; HPAIV: highly pathogenic avian influenza virus; kb: kilobase pairs; LPAIV: low pathogenic avian influenza virus; Mb: megabase pairs; NCBI: National Center for Biotechnology Information; NGS: next-generation sequencing; nt: nucleotides; ORF: open reading frame; PacBio: Pacific Biosciences; SRA: Sequence Read Archive; TAMA: Transcriptome Annotation by Modular Algorithms; VGL: Vertebrate Genomes Lab; VGP: Vertebrate Genomes Project; ZMW: zero-mode waveguide.
