# Supplementary material for: A high-quality genome and comparison of short- versus long-read transcriptome of the palaearctic duck Aythya fuligula (tufted duck)
Source: Gigascience. 2021 Dec 20;10(12):giab081. doi: 10.1093/gigascience/giab081 (PMC8685854; doi:10.1093/gigascience/giab081)
Supplement: giab081_Supplemental_Files [file giab081_supplemental_files.zip › 20211012_rmueller_tufted_duck_supp.pdf]

Supplementary data for "A high-quality Genome and Comparison of Short versus Long Read Transcriptome of the Palaearctic duck *Aythya fuligula* (Tufted Duck)"

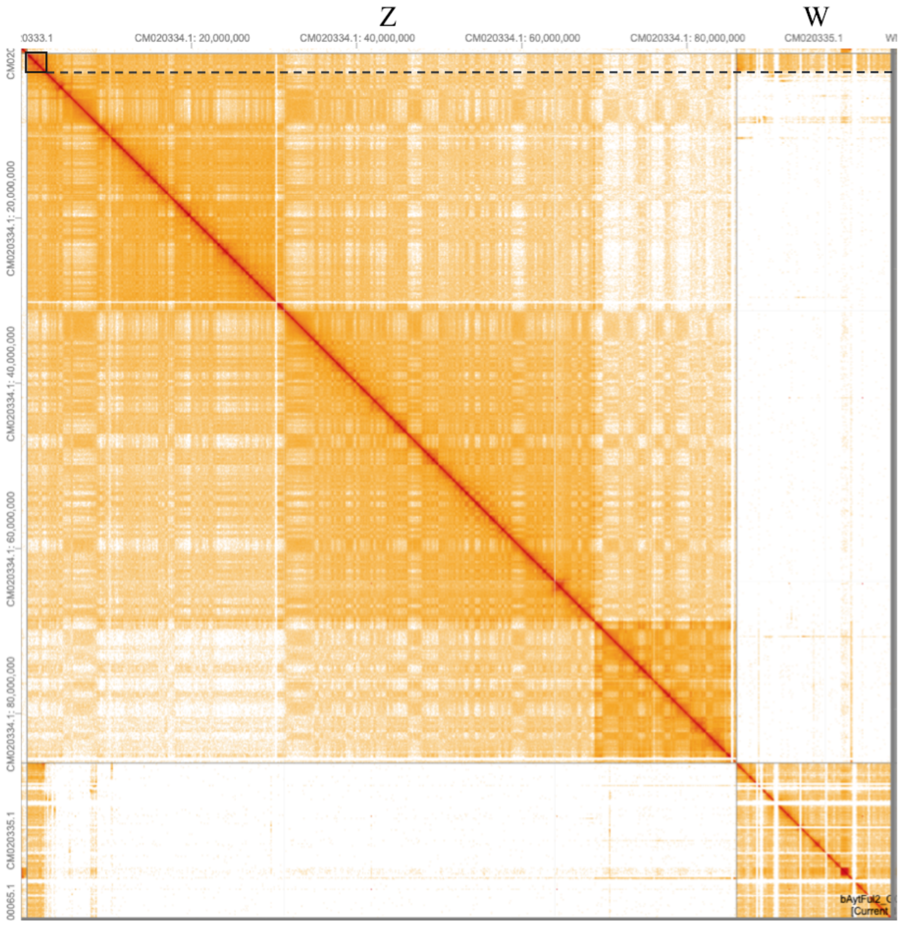

Figure S1. Hi-C contact map of *Aythya fuligula* visualised in HiGlass. The plot shows the interaction in the chromatin space of chromosomes Z and W. The shading indicates the level of interaction occurring along the chromosomes: the more closely interacting, the greater the degree of shading. This Hi-C map shows the near-complete separation of the sex chromosomes, except for the contact interaction in the 2.1 Mb pseudoautosomal region (black box), for which there is only a single representation in the assembly of the Z chromosome.

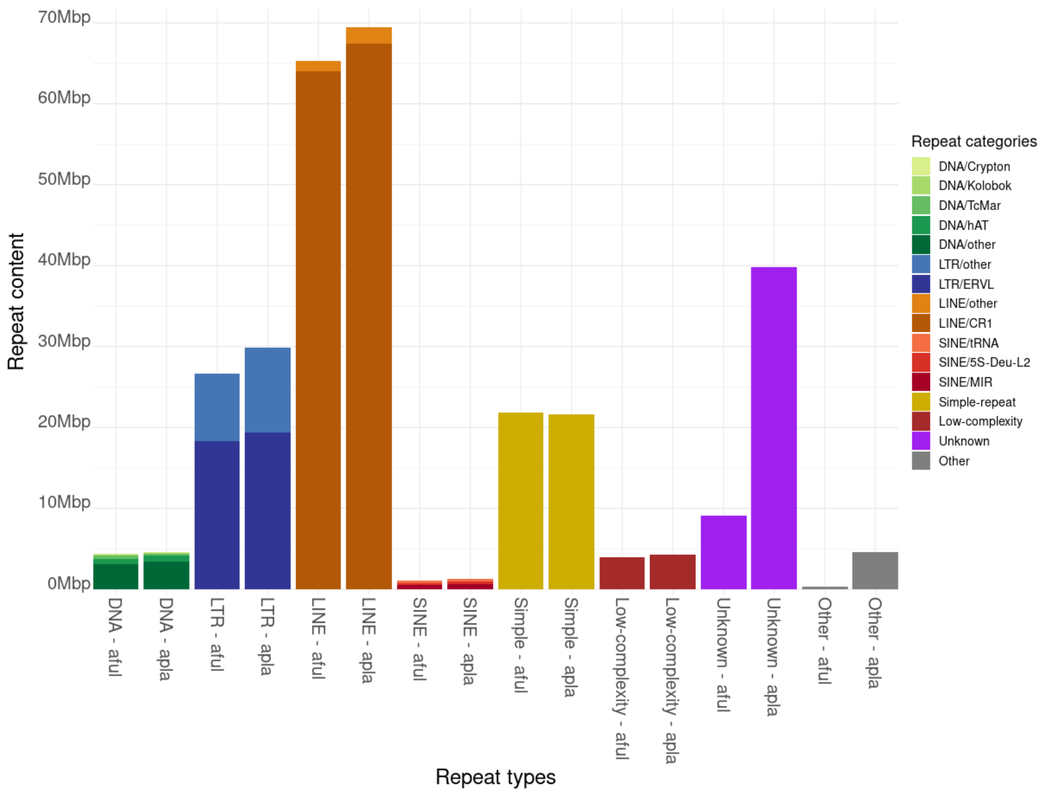

Figure S2. Comparison of genomic repeat content between tufted duck (bAytFul2.pri) and the mallard (ZJU1.0). Repeat content is shown for the major repeat classes and within each class the major repeat types are also shown.

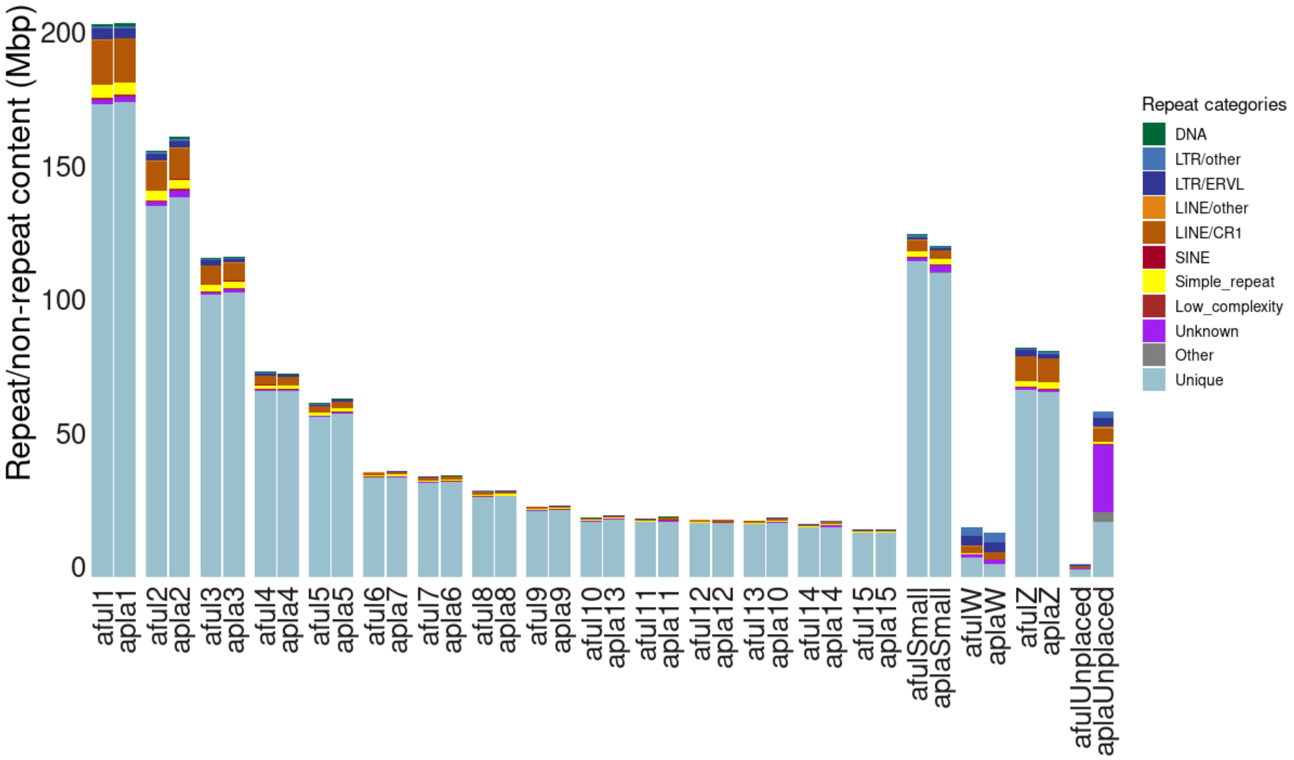

Figure S3. Comparison of repeat and unique sequence content in orthologous chromosome pairs between tufted duck (aful) and mallard (apla). Repeat content is shown for individual chromosomes for chr1 to chr15. Repeat content in the smaller chromosomes are shown under afulSmall and aplaSmall by merging together the sequences of the smaller chromosomes. Repeat content in the unplaced sequences are shown in afulUnplaced and aplaUnplaced.

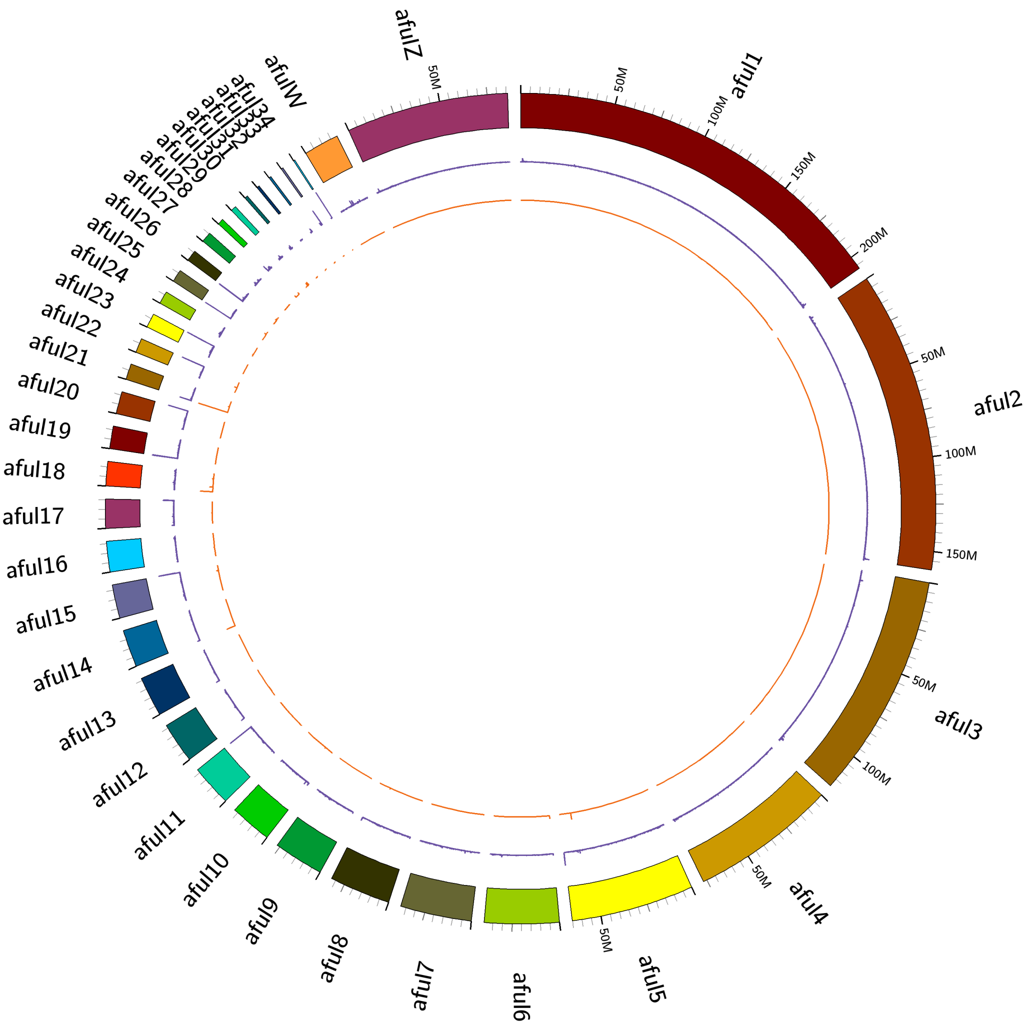

Figure S4. Identified telomeric and centromeric repeats in the tufted duck genome assembly. Frequency of telomere and centromere specific repeats were calculated in 10kb windows.

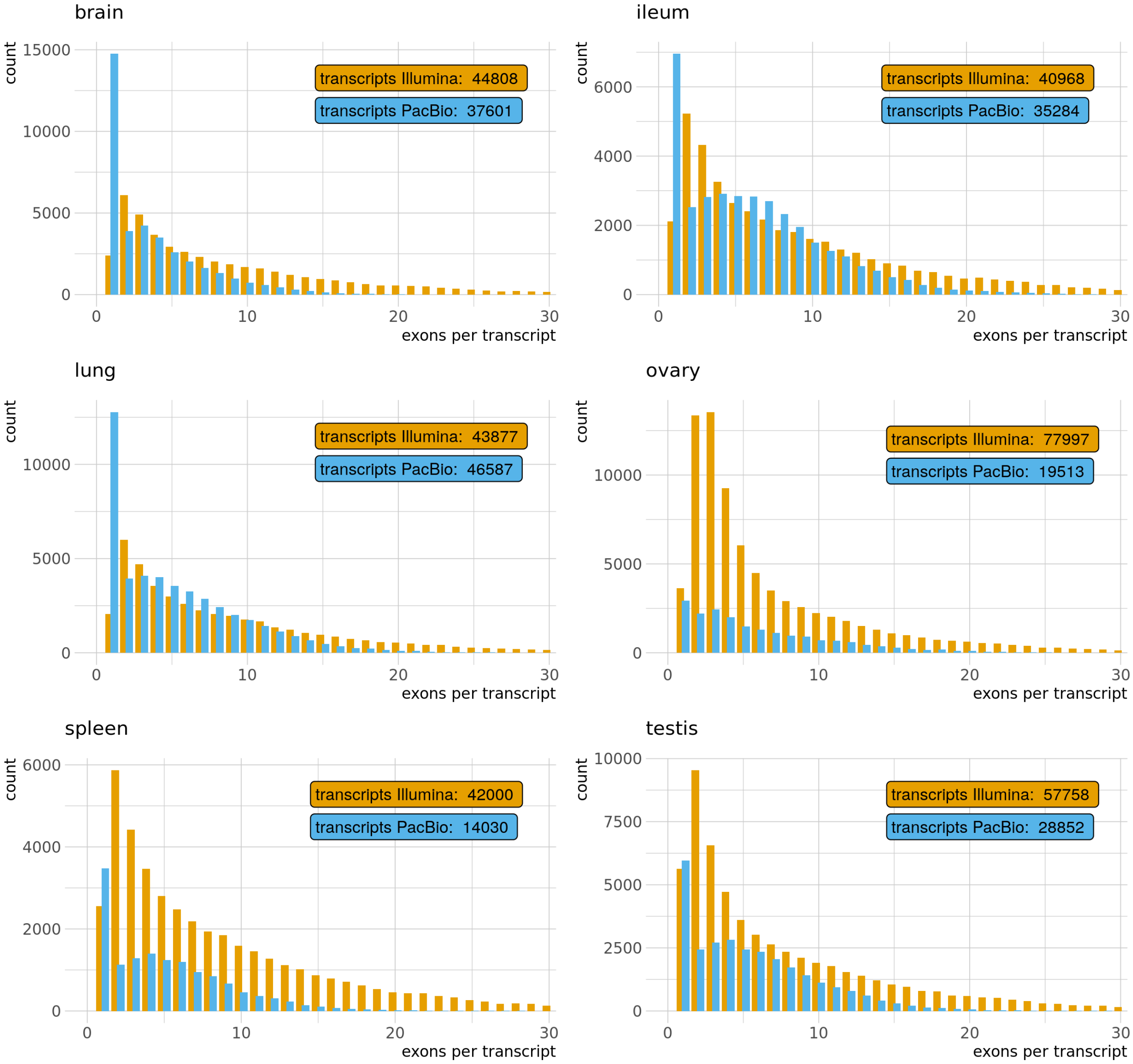

Figure S5. Distribution of single-and multi-exon transcripts per tissue and pipeline. Only the first 30 groups are shown.

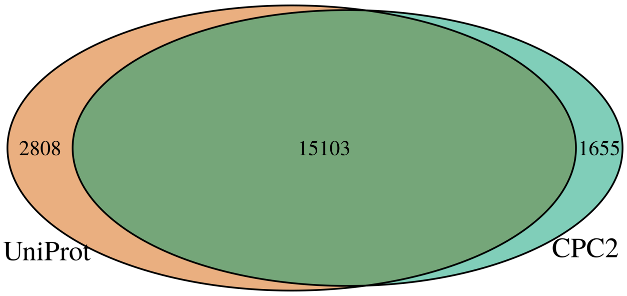

Figure S6. Protein-coding potential calculated by CPC2 intersected with hits against the UniRef50 database (merged transcripts of all tissues and pipelines).

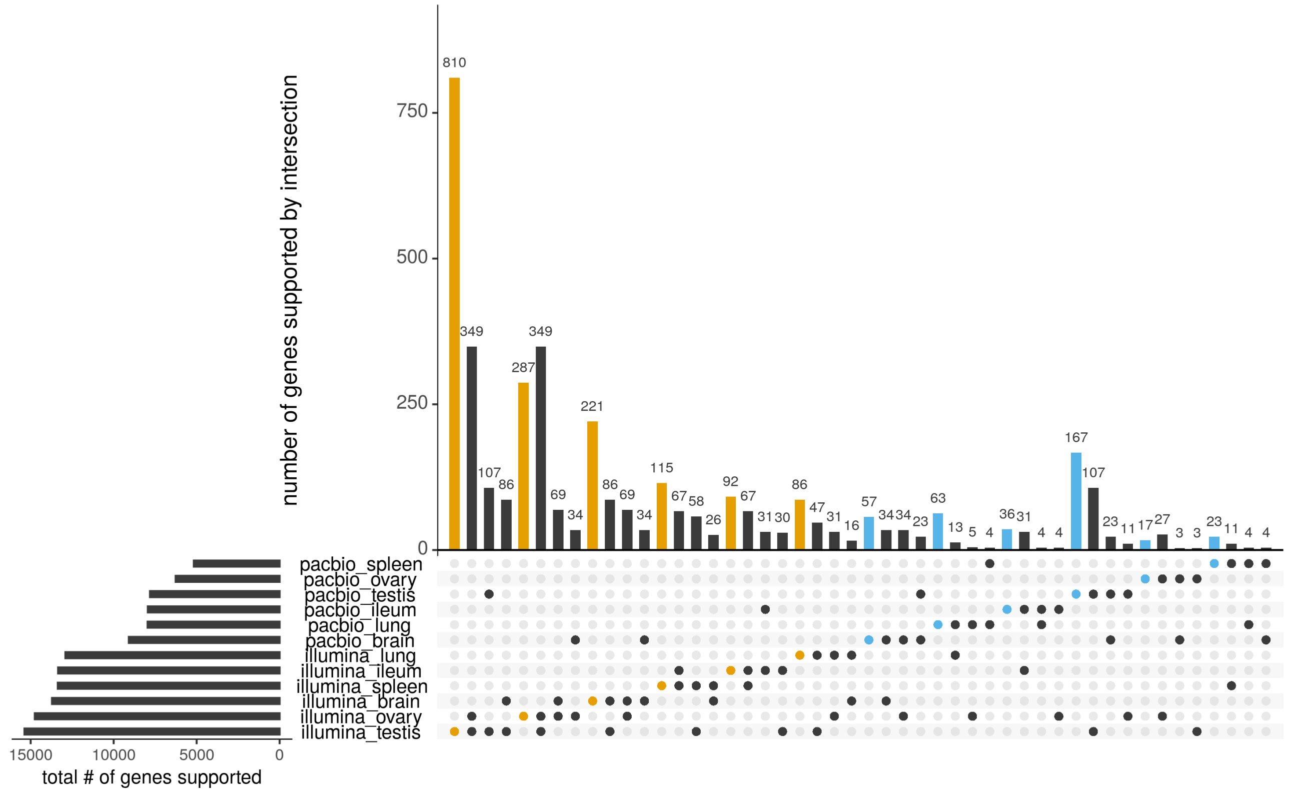

Figure S7. Pipeline-tissue-specific expression of genes found in UniProt: Tissue-exclusive Illumina genes marked yellow, tissue-exclusive PacBio genes marked blue. Bottom left panel: total number of identified genes by pipeline and tissue ordered by the number of identified genes. Main panel: 12 grouped sets (pipeline-tissue) with exclusive genes plus top three intersections with other pipeline or tissue. The number of annotated genes was generally higher with Illumina transcriptome reconstruction than with that of PacBio. In both pipelines, testis expressed the highest number of genes exclusively, with 810 in Illumina data and 167 from PacBio.

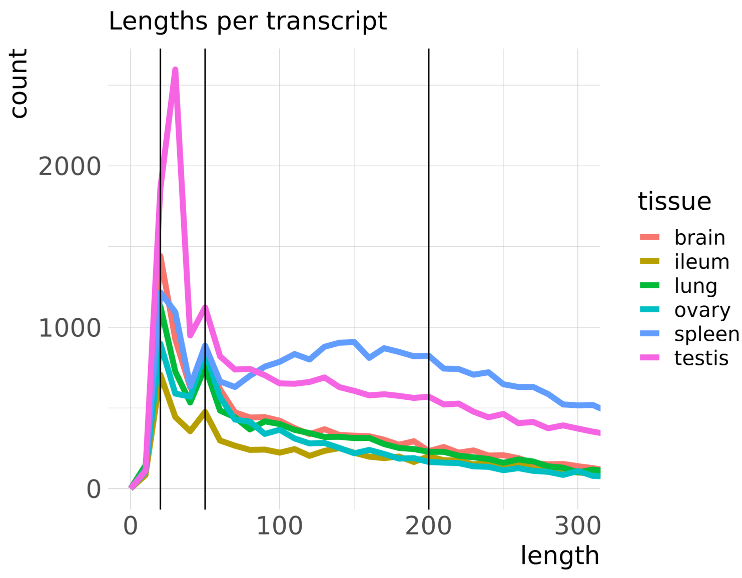

Figure S8. Length distribution of small RNA transcripts follow decaying function with peaks at 20 bp (30 bp in testis) and 50 bp. Vertical bars at 20 bp, 50 bp and 200 bp were added.

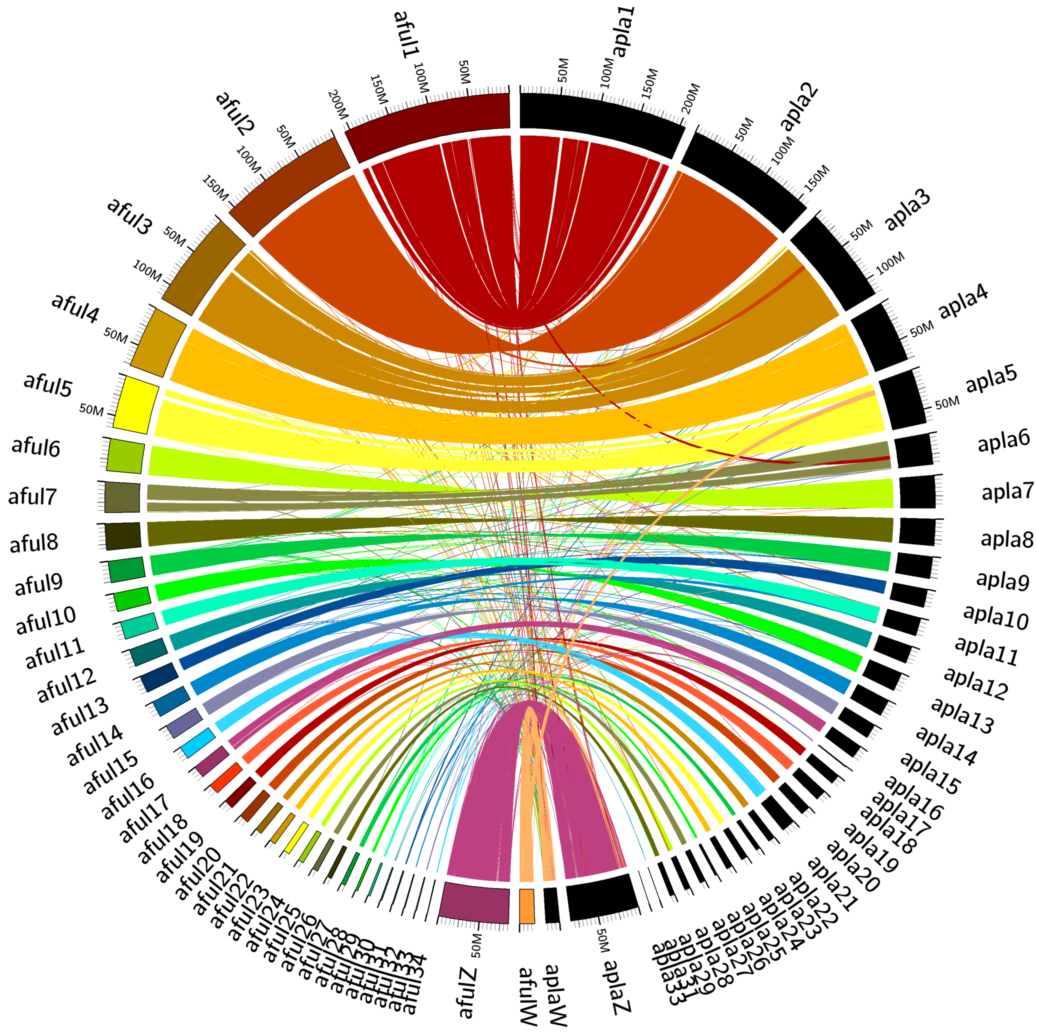

Figure S9. Orthologous chromosome pairs of tufted duck (aful) and mallard (apla).

Table S1. Assembly metrics before and after curation (excluding mitochondrion). Karyotype is 39 chromosome pairs, while 34 autosomes plus Z and W chromosomes were identified. Total length: 1,126,988,109 bp; assigned to chromosomes: 1,122,119,279 bp (99.56%).

Assembly metrics		before	after
scaffolds	total [bp]	1,136,615,867	1,126,988,109
	count	123	104
	N50 [bp]	72,361,183	85,905,639
	L50	4	4
	N90 [bp]	12,903,048	15,484,054
	L90	18	19
contigs	total [bp]	1,117,782,556	1,117,570,712
	count	270	266
	N50 [bp]	17,816,505	17,816,505
	L50	19	19
	N90 [bp]	4,225,905	4,225,905
	L90	65	65

Table S2. Assembly and annotation statistics. Columns 1–10 from https://www.ncbi.nlm.nih.gov/assembly/GCF_009819795.1#/def_asm_Primary_Assembly, %GC content after curation, predicted and annotated genes (after conservative filtering) per scaffold from the final annotation. Table ordered by chromosomes (named based on descending size) plus unplaced scaffolds (aggregated) and mitochondrion.

Molecule name	GenBank sequence	RefSeq sequence	Unlocalized sequences count	Total length [bp]	Scaffold count	Ungapped length [bp]	Scaffold N50 [bp]	Spanned gaps	Unspanned gaps	%GC content	Predicted genes	Annotated genes
Chromosome 1	CM020300.1	NC_045559.1	0	207,018,403	1	206,331,808	207,018,403	17	0	40.00	7,790	2,053
Chromosome 2	CM020301.1	NC_045560.1	0	159,650,012	1	158,529,885	159,650,012	16	0	39.74	5,723	1,335
Chromosome 3	CM020302.1	NC_045561.1	0	119,491,689	1	119,467,459	119,491,689	8	0	40.07	4,698	1,141
Chromosome 4	CM020303.1	NC_045562.1	0	76,878,203	1	76,505,370	76,878,203	8	0	39.67	2,875	729
Chromosome 5	CM020304.1	NC_045563.1	0	65,067,180	1	64,714,765	65,067,180	4	0	41.28	2,886	846
Chromosome 6	CM020305.1	NC_045564.1	0	39,514,636	1	39,452,091	39,514,636	1	0	41.08	1,659	420
Chromosome 7	CM020306.1	NC_045565.1	0	37,840,942	1	37,637,229	37,840,942	3	0	41.60	1,573	454
Chromosome 8	CM020307.1	NC_045566.1	0	32,406,295	1	32,406,269	32,406,295	2	0	41.88	1,449	440
Chromosome 9	CM020308.1	NC_045567.1	0	26,499,672	1	26,470,423	26,499,672	2	0	42.73	1,283	363
Chromosome 10	CM020309.1	NC_045568.1	0	22,162,769	1	22,162,769	22,162,769	0	0	43.16	1,072	298
Chromosome 11	CM020310.1	NC_045569.1	0	21,928,510	1	21,928,510	21,928,510	0	0	43.10	1,036	334
Chromosome 12	CM020311.1	NC_045570.1	0	21,526,058	1	21,526,058	21,526,058	0	0	42.11	944	311
Chromosome 13	CM020312.1	NC_045571.1	0	21,228,795	1	21,173,751	21,228,795	2	0	42.66	1,056	301
Chromosome 14	CM020313.1	NC_045572.1	0	19,924,658	1	19,924,658	19,924,658	0	0	44.47	990	292
Chromosome 15	CM020314.1	NC_045573.1	0	17,816,505	1	17,816,505	17,816,505	0	0	44.63	949	343
Chromosome 16	CM020315.1	NC_045574.1	0	16,262,610	1	16,129,940	16,262,610	3	0	45.62	951	303
Chromosome 17	CM020316.1	NC_045575.1	0	15,484,054	1	15,373,460	15,484,054	2	0	45.48	881	299
Chromosome 18	CM020317.1	NC_045576.1	0	12,903,739	1	12,903,339	12,903,739	2	0	46.89	780	243
Chromosome 19	CM020318.1	NC_045577.1	0	11,953,284	1	11,953,084	11,953,284	1	0	48.04	699	257
Chromosome 20	CM020319.1	NC_045578.1	0	11,861,279	1	11,861,279	11,861,279	0	0	46.78	657	259
Chromosome 21	CM020320.1	NC_045579.1	0	8,424,528	1	8,424,528	8,424,528	0	0	47.64	550	203
Chromosome 22	CM020321.1	NC_045580.1	0	7,582,326	1	7,582,326	7,582,326	0	0	48.80	487	138
Chromosome 23	CM020322.1	NC_045581.1	0	7,507,630	1	7,507,630	7,507,630	0	0	50.44	494	192
Chromosome 24	CM020323.1	NC_045582.1	0	6,710,917	1	6,501,815	6,710,917	4	0	51.08	539	228
Chromosome 25	CM020324.1	NC_045583.1	0	6,693,931	1	6,693,931	6,693,931	0	0	51.92	540	215
Chromosome 26	CM020325.1	NC_045584.1	0	5,835,185	1	5,761,714	5,835,185	5	0	52.33	500	232
Chromosome 27	CM020326.1	NC_045585.1	0	5,453,253	1	5,452,853	5,453,253	2	0	48.27	397	145
Chromosome 28	CM020327.1	NC_045586.1	0	3,167,070	1	2,925,066	3,167,070	4	0	56.40	357	171
Chromosome 29	CM020328.1	NC_045587.1	0	2,844,608	1	2,688,101	2,844,608	7	0	56.04	314	129
Chromosome 30	CM020329.1	NC_045588.1	0	1,560,659	1	731,800	1,560,659	3	0	53.60	103	46
Chromosome 31	CM020330.1	NC_045589.1	0	1,405,878	1	1,305,761	1,405,878	3	0	49.89	87	17
Chromosome 32	CM020331.1	NC_045590.1	0	1,164,621	1	1,164,608	1,164,621	1	0	50.72	122	38
Chromosome 33	CM020332.1	NC_045591.1	0	983,965	1	982,965	983,965	2	0	52.89	117	54
Chromosome 34	CM020333.1	NC_045592.1	0	723,082	1	723,082	723,082	0	0	50.02	42	11
Chromosome Z	CM020334.1	NC_045593.1	0	85,905,639	1	84,574,782	85,905,639	20	0	40.05	2,878	772
Chromosome W	CM020335.1	NC_045594.1	0	18,736,694	1	15,551,531	18,736,694	21	0	46.44	1,506	329
unplaced	n/a	n/a	68	4,868,830	68	4,729,567	120,298	20	0	54.07	751	154
Mitochondrion MT	KJ722069.1	NC_024595.1		16,616	1	16,616	n/a	n/a	n/a	n/a	11	4
Sums (AVG for %GC)			68	1,127,004,725	105	1,117,587,328	n/a	163	0	46.53	49,746	14,099

Table S3. Gene prediction and transcript model reconstruction by pipeline and tissue of the ORFs matching the mallard RIG-I gene.

Protein	Gene	Predicted in pipeline (tissue)	Transcript	Reconstructed in pipeline (tissue)
IFIH1	G24916	Illumina (all tissues), PacBio (brain, ileum, lung, testis)	G24916.1<br>G24916.2<br>G24916.3<br>G24916.4<br>G24916.7<br>G24916.8	Illumina (spleen, testis)<br>Illumina (spleen, testis)<br>Illumina (brain)<br>Illumina (brain, lung, ovary)<br>Illumina (ileum)<br>Illumina (ileum)
RIG-I/DDX58	G46857	Illumina (all tissues), PacBio (brain, ileum, lung)	G46857.2<br>G46857.3<br>G46857.4	Illumina (brain, lung)<br>Illumina (ileum, ovary, spleen, testis)<br>PacBio (lung)

Table S4. Protein variation effect analysis of 14 substitutions and one insertion with PROVEAN predicted no impact of the varying sites in the tufted duck RIG-I/DDX58 gene on biological function.

Variant	PROVEAN score	Prediction (cutoff = -2.5)
S8G	-0.817	Neutral
R180S	0.660	Neutral
V240A	-0.149	Neutral
S400_A401insS	2.222	Neutral
V616A	1.092	Neutral
P677L	-2.167	Neutral
T845I	0.851	Neutral
D4E	0.619	Neutral
S126N	-0.581	Neutral
N259D	0.385	Neutral
T362S	-0.225	Neutral
A475V	-1.016	Neutral
L497V	-1.106	Neutral
N655S	0.336	Neutral
K768E	-1.452	Neutral

Table S5. According to SIFT, all amino acid substitutions are predicted to be tolerated in the varying sites in the tufted duck RIG-I/DDX58 gene.

Position	Substitution	Prediction	Score	Median sequence conservation	Sequences represented at this position
4	D to E	TOLERATED	1.00	3.57	4
8	S to G	TOLERATED	0.26	3.57	4
126	S to N	TOLERATED	0.26	3.39	5
180	R to S	TOLERATED	0.12	3.39	5
240	V to A	TOLERATED	0.55	3.35	6
259	N to D	TOLERATED	0.58	3.05	8
362	T to S	TOLERATED	0.49	3.05	8
475	A to V	TOLERATED	0.56	3.39	6
497	L to V	TOLERATED	0.12	3.11	7
616	V to A	TOLERATED	0.58	3.05	8
655	N to S	TOLERATED	0.92	3.05	8
677	P to L	TOLERATED	0.24	3.05	8
768	K to E	TOLERATED	0.86	3.05	8
845	T to I	TOLERATED	0.73	3.05	8

Table S6. Transcription factor binding sites 1 kb upstream of the RIG-I/DDX58 gene are identical in mallard and tufted duck according to P-Match.

Matrix identifier	Position (strand)	Core d-score	Matrix d-score	Sequence (always the (+)-strand is shown)	Factor name
V$NKX25_02	63 (+)	1.000	1.000	caTAATTa	Nkx2-5
V$NFKAPPAB65_01	331 (-)	1.000	1.000	GGAAAttccc	NF-kappaB (p65)
V$CREL_01	331 (-)	1.000	1.000	GGAAAttccc	c-Rel
V$NFKAPPAB_01	331 (-)	1.000	1.000	ggaaaTTCCC	NF-kappaB

Table S7. RNA extraction

Sample	Weight [mg]	Concentration [ng/µL]	RIN
TD_brain_52037	123	202.7	9.2
TD_ileum_52037	42	958.2	10.0
TD_lung_52037	37	221.6	9.5
TD_ovary_52037	55	1037.4	9.5
TD_spleen_52037	53	2109.8	9.4
TD_testis_54068	63	989.3	9.7

Table S8. small RNA extraction

Sample	Weight [mg]	Concentration [ng/µL]	RIN
TD_brain_F52037	29	852.87	9.3
TD_ileum_F52037	16	727.93	10.0
TD_lung_F50325	15	402.63	9.7
TD_ovary_F50325	12	368.79	9.4
TD_spleen_F50325	5	823.70	9.5
TD_testis_M54068	19	722.62	9.4

Table S9. cDNA libraries for Iso-Seq

Sample	Concentration [ng/µL]
TD_brain_52037	46.2
TD_ileum_52037	43.2
TD_lung_52037	33.2
TD_ovary_52037	41.0
TD_spleen_52037	66.8
TD_testis_54068	45.2

Listing 1. Tblastn result of Query: antiviral innate immune response receptor RIG-I [Anas platyrhynchos] Query ID: NP_001297309.1 Length: 933.

```
>PREDICTED: Aythya fuligula DExD/H-box helicase 58 (DDX58), mRNA
Sequence ID: XM_032205362.1 Length: 5179
Range 1: 329 to 3130

Score:1882 bits(4875), Expect:0.0,
Method:Compositional matrix adjust.,
Identities:918/934(98%), Positives:926/934(99%), Gaps:1/934(0%)

Query  1     MTADEKRSLQCYRRYIERSLNPVYVLGNMTDWLPDelrerirkeeerGVSGAAALFLDAV  60
             MTA+EKR LQCYRRYIERSLNPVYVLGNMTDWLPDELRERIRKEEERGVSGAAALFLDAV
Sbjct  329   MTAEEKRGLQCYRRYIERSLNPVYVLGNMTDWLPDELRERIRKEEERGVSGAAALFLDAV  508

Query  61    LQLEARGWFRGMLDAMLAAGYTGLAEAIENWDFSKLEKLELHRQLLKRIEATMLEVDPVA  120
             LQLEARGWFRGMLDAMLAAGYTGLAEAIENWDFSKLEKLELHRQLLKRIEATMLEVDPVA
Sbjct  509   LQLEARGWFRGMLDAMLAAGYTGLAEAIENWDFSKLEKLELHRQLLKRIEATMLEVDPVA  688

Query  121   LIPYISTCLIDRECEEIQQISENRSKAAGITKLIECLCRSDKEHWPKSLQLALDTTGYYR  180
             LIPYI+TCLIDRECEEIQQISENRSKAAGITKLIECLCRSDKEHWPKSLQLALDTTGYY
Sbjct  689   LIPYINTCLIDRECEEIQQISENRSKAAGITKLIECLCRSDKEHWPKSLQLALDTTGYYS  868

Query  181   ASELWDIREDNAKDVDSEMTDASEDCLEASMTYSEEAEPDDNLSENLGSAAEGIGKPPPV  240
             ASELWDIREDNAKDVDSEMTDASEDCLEASMTYSEEAEPDDNLSENLGSAAEGIGKPPP
Sbjct  869   ASELWDIREDNAKDVDSEMTDASEDCLEASMTYSEEAEPDDNLSENLGSAAEGIGKPPPA  1048

Query  241   YETKKARSYQIELAQPAINGKNALICAPTGSGKTFVSILICEHHFQNMPAGRKAKVVFLA  300
             YETKKARSYQIELAQPAI+GKNALICAPTGSGKTFVSILICEHHFQNMPAGRKAKVVFLA
Sbjct  1049  YETKKARSYQIELAQPAIDGKNALICAPTGSGKTFVSILICEHHFQNMPAGRKAKVVFLA  1228

Query  301   TKVPVYEQQKNVFKHHFERQGYSVQGISGENFSNVSVEKVIEDSDIIVVTPQILVNSFED  360
             TKVPVYEQQKNVFKHHFERQGYSVQGISGENFSNVSVEKVIEDSDIIVVTPQILVNSFED
Sbjct  1229  TKVPVYEQQKNVFKHHFERQGYSVQGISGENFSNVSVEKVIEDSDIIVVTPQILVNSFED  1408

Query  361   GTLTSLSIFTLMIFDECHNTTGNHPYNVLMTRYLEQKFNS-ASQLPQILGLTASVGVGNA  419
             G+LTSLSIFTLMIFDECHNTTGNHPYNVLMTRYLEQKFNS ASQLPQILGLTASVGVGNA
Sbjct  1409  GSLTSLSIFTLMIFDECHNTTGNHPYNVLMTRYLEQKFNSSASQLPQILGLTASVGVGNA  1588

Query  420   KNIEETIEHICSLCSYLDIQAISTVRENIQELQRFMNKPEIDVRLVKRRIHNPFAAIISN  479
             KNIEETIEHICSLCSYLDIQAISTVRENIQELQRFMNKPEIDVRLVKRR+HNPFA IISN
Sbjct  1589  KNIEETIEHICSLCSYLDIQAISTVRENIQELQRFMNKPEIDVRLVKRRVHNPFAVIISN  1768

Query  480   LMSETEALMRTIYSVDTLSQNSKKDFGTQNYEHWIVVTQRKCRLLQLEDKEEESRICRAL  539
             LMSETEALMRTIYSVDT+SQNSKKDFGTQNYEHWIVVTQRKCRLLQLEDKEEESRICRAL
Sbjct  1769  LMSETEALMRTIYSVDTVSQNSKKDFGTQNYEHWIVVTQRKCRLLQLEDKEEESRICRAL  1948

Query  540   FICTEHLRKYNDALIISEDARIIDALSYLTEFFTNVKNGPYTELEQHLTAKFQEKEPELI  599
             FICTEHLRKYNDALIISEDARIIDALSYLTEFFTNVKNGPYTELEQHLTAKFQEKEPELI
Sbjct  1949  FICTEHLRKYNDALIISEDARIIDALSYLTEFFTNVKNGPYTELEQHLTAKFQEKEPELI  2128

Query  600   ALSKDETNENPKLEELVCILDDAYRYNPQTRTLLFAKTRALVSALKKCMEENPILNYIKP  659
             ALSKDETNENPKLEEL CILDDAYRYNPQTRTLLFAKTRALVSALKKCMEENPIL+YIKP
Sbjct  2129  ALSKDETNENPKLEELACILDDAYRYNPQTRTLLFAKTRALVSALKKCMEENPILSYIKP  2308

Query  660   GVLMGRGRRDQTTGMTLPSQKGVLDAFKTSKDNRLLIATSVADEGIDIVQCNLVVLYEYS  719
             GVLMGRGRRDQTTGMTL SQKGVLDAFKTSKDNRLLIATSVADEGIDIVQCNLVVLYEYS
Sbjct  2309  GVLMGRGRRDQTTGMTLLSQKGVLDAFKTSKDNRLLIATSVADEGIDIVQCNLVVLYEYS  2488

Query  720   GNVTKMIQVRGRGRAAGSKCILVTSKTEVVENEKCNRYKEEMMNKAVEKIQKWDEETFAK  779
             GNVTKMIQVRGRGRAAGSKCILVTSKTEVVENEKCNRYKEEMMNKAVE+IQKWDEETFAK
Sbjct  2489  GNVTKMIQVRGRGRAAGSKCILVTSKTEVVENEKCNRYKEEMMNKAVEEIQKWDEETFAK  2668

Query  780   KIHNLQMKERVLRDSRRKEIKPKVVEGQKNLLCGKCKAYACSTDDIRIIKDSHHIVLGEA  839
             KIHNLQMKERVLRDSRRKEIKPKVVEGQKNLLCGKCKAYACSTDDIRIIKDSHHIVLGEA
Sbjct  2669  KIHNLQMKERVLRDSRRKEIKPKVVEGQKNLLCGKCKAYACSTDDIRIIKDSHHIVLGEA  2848

Query  840   FKERYTTKPHKKPMQFDGFEKKSKMYCRNNNCQHDWGITVKYLTFDNLPVIKIKSFVMES  899
             FKERY TKPHKKPMQFDGFEKKSKMYCRNNNCQHDWGITVKYLTFDNLPVIKIKSFVMES
Sbjct  2849  FKERYITKPHKKPMQFDGFEKKSKMYCRNNNCQHDWGITVKYLTFDNLPVIKIKSFVMES  3028

Query  900   TATGTQMDFQKWKSINSSLKNFDVEEMSNLYPPF  933
             TATGTQMDFQKWKSINSSLKNFDVEEMSNLYPPF
Sbjct  3029  TATGTQMDFQKWKSINSSLKNFDVEEMSNLYPPF  3130.
```
