# Supplementary figures and images for: A high-quality genome and comparison of short- versus long-read transcriptome of the palaearctic duck Aythya fuligula (tufted duck)
Source: Gigascience. 2021 Dec 20;10(12):giab081. doi: 10.1093/gigascience/giab081 (PMC8685854; doi:10.1093/gigascience/giab081)

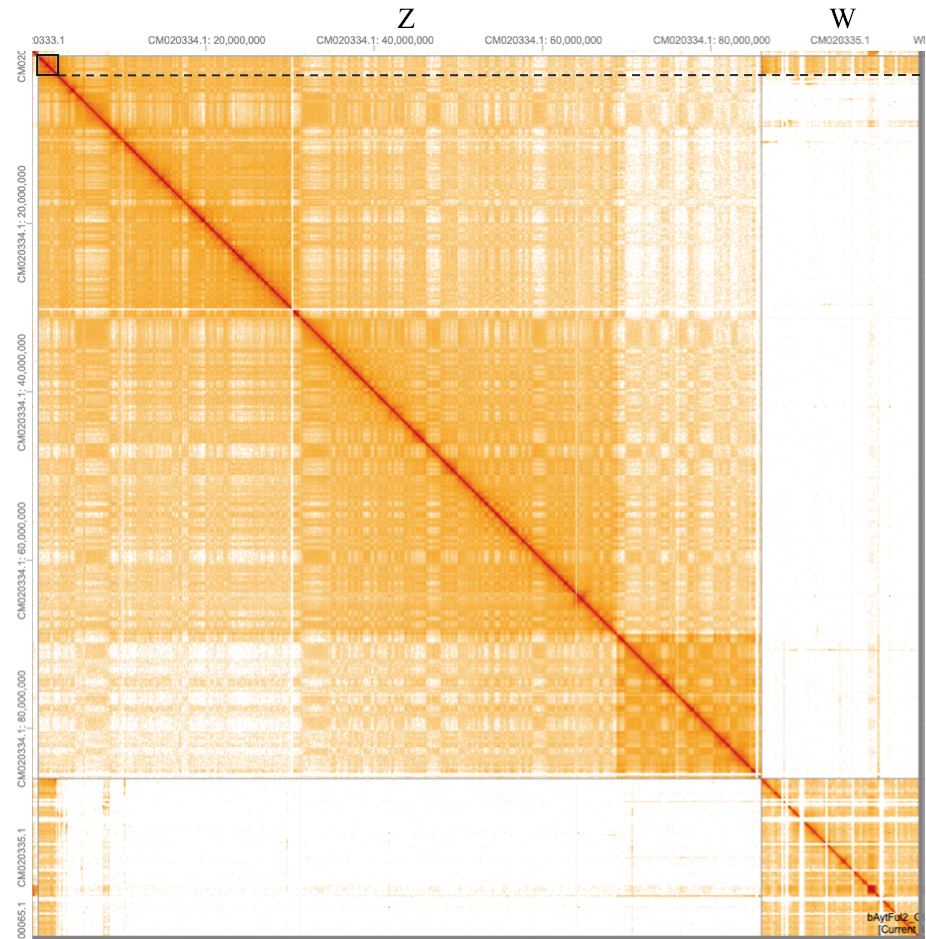

Supplement: giab081_Supplemental_Files [file giab081_supplemental_files.zip › s1_bAytFul2_Z_W.png]

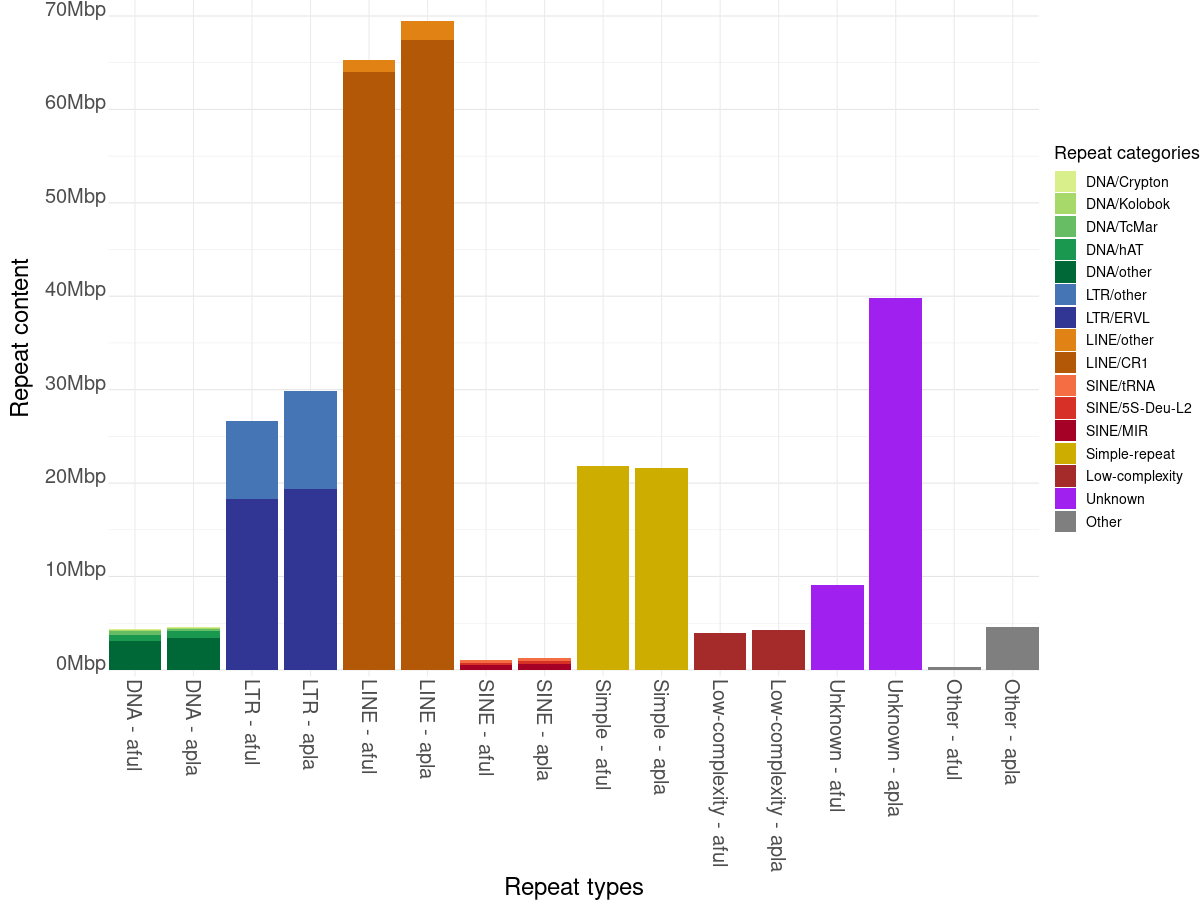

Supplement: giab081_Supplemental_Files [file giab081_supplemental_files.zip › s2_repeat_composition_tufted_mallard.png]

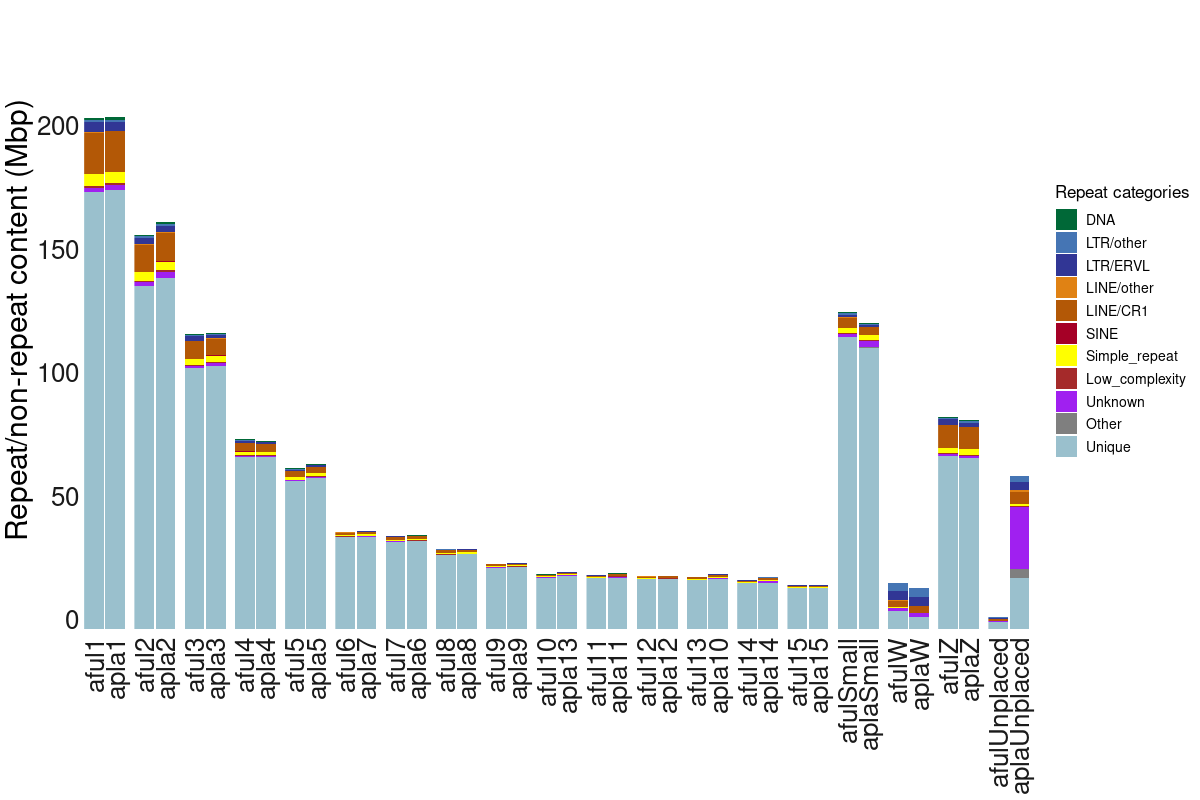

Supplement: giab081_Supplemental_Files [file giab081_supplemental_files.zip › s3_tufted_mallard_repeat_contet_per_chr.png]

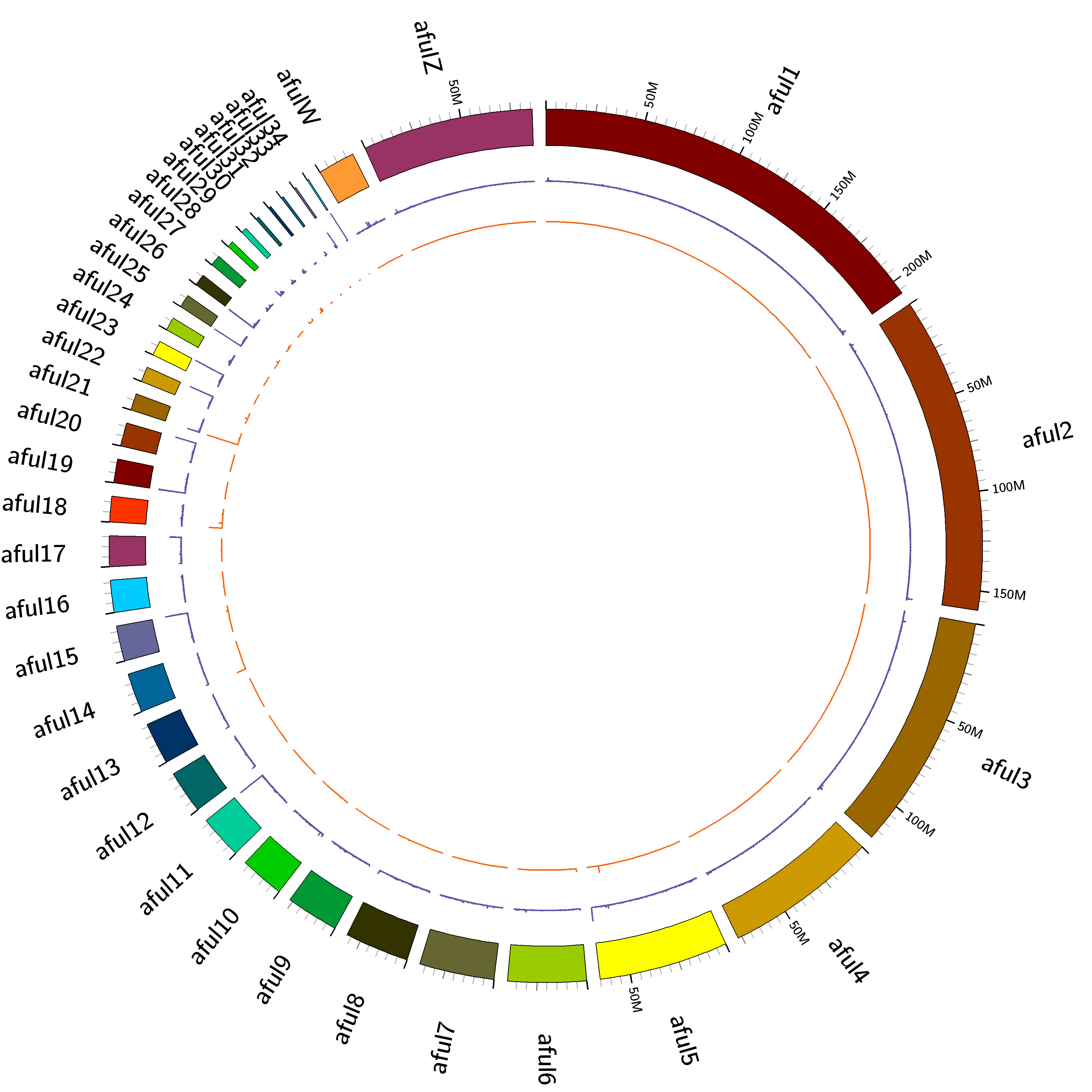

Supplement: giab081_Supplemental_Files [file giab081_supplemental_files.zip › s4_tufted_telomere_centromere.png]

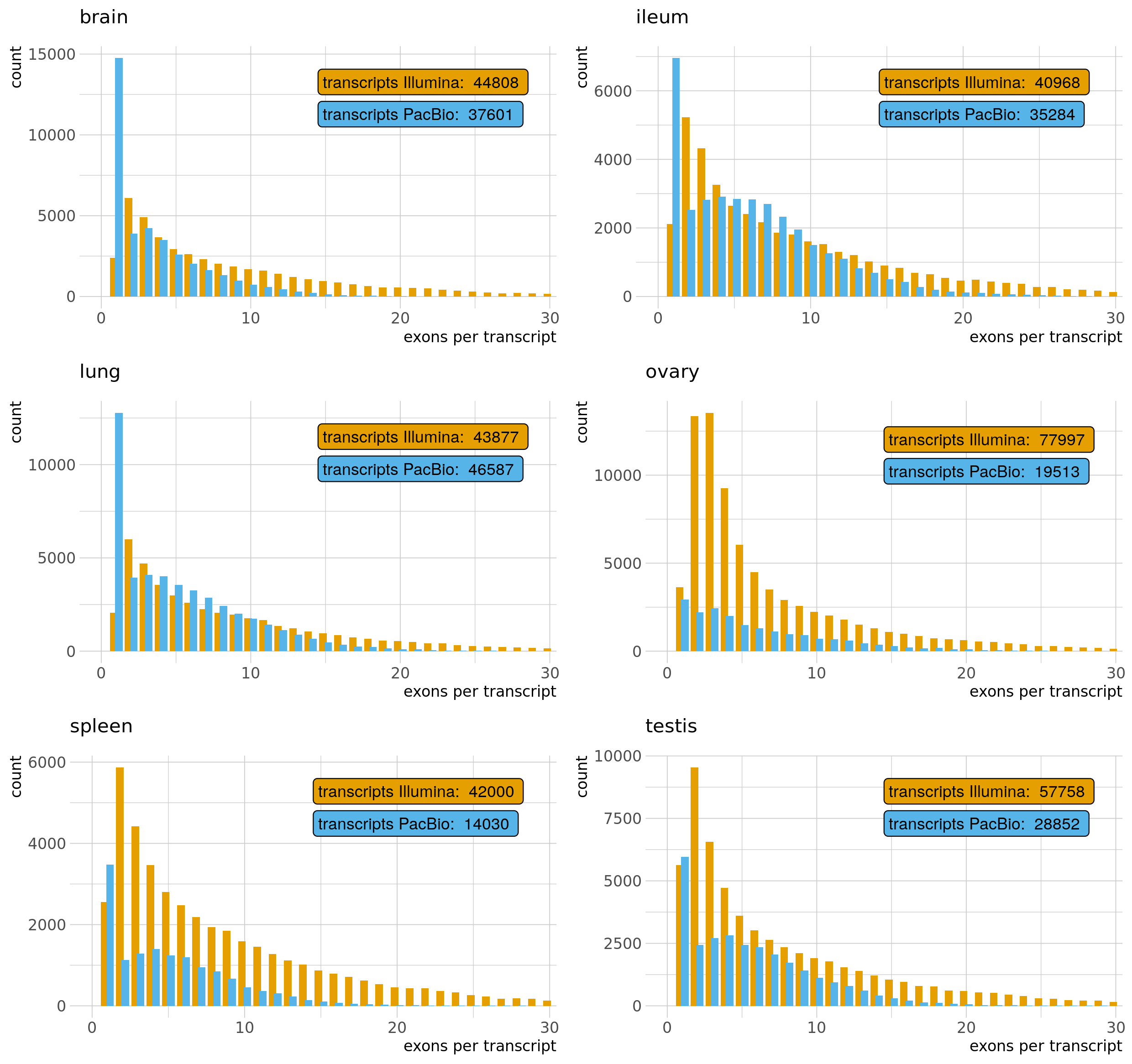

Supplement: giab081_Supplemental_Files [file giab081_supplemental_files.zip › s5_exons_trans.png]

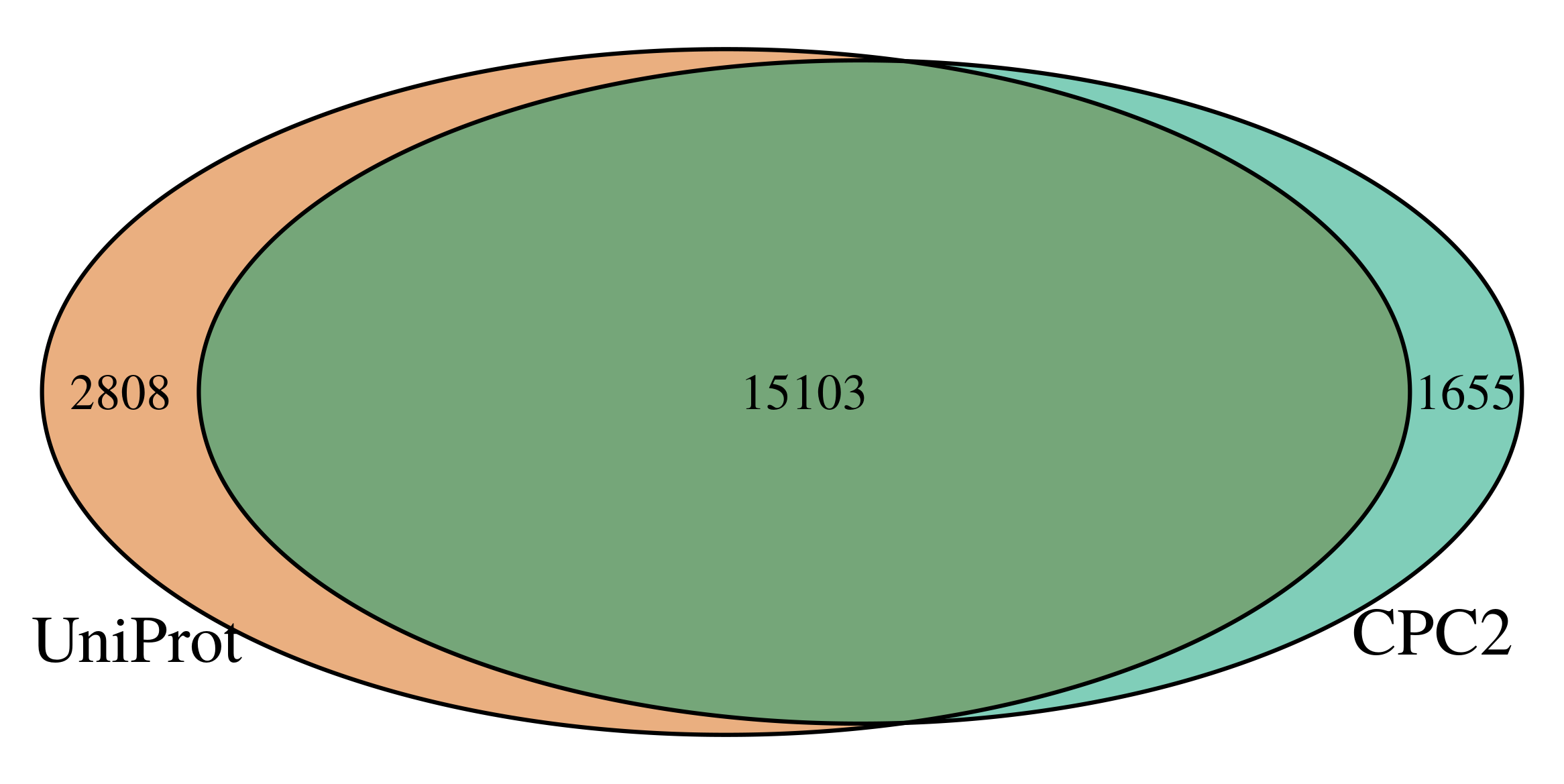

Supplement: giab081_Supplemental_Files [file giab081_supplemental_files.zip › s6_coding_potential.png]

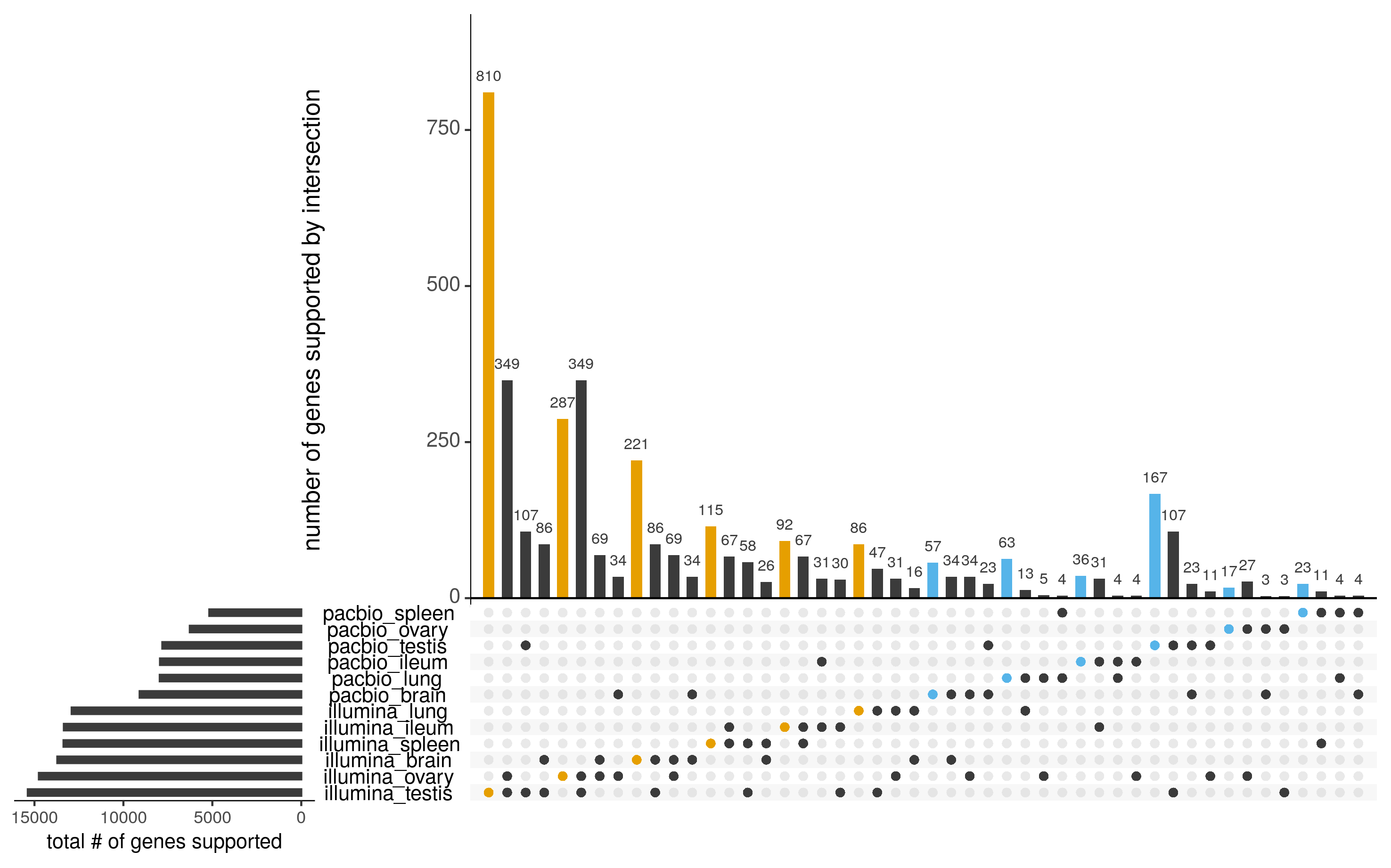

Supplement: giab081_Supplemental_Files [file giab081_supplemental_files.zip › s7_intersect_plot.png]

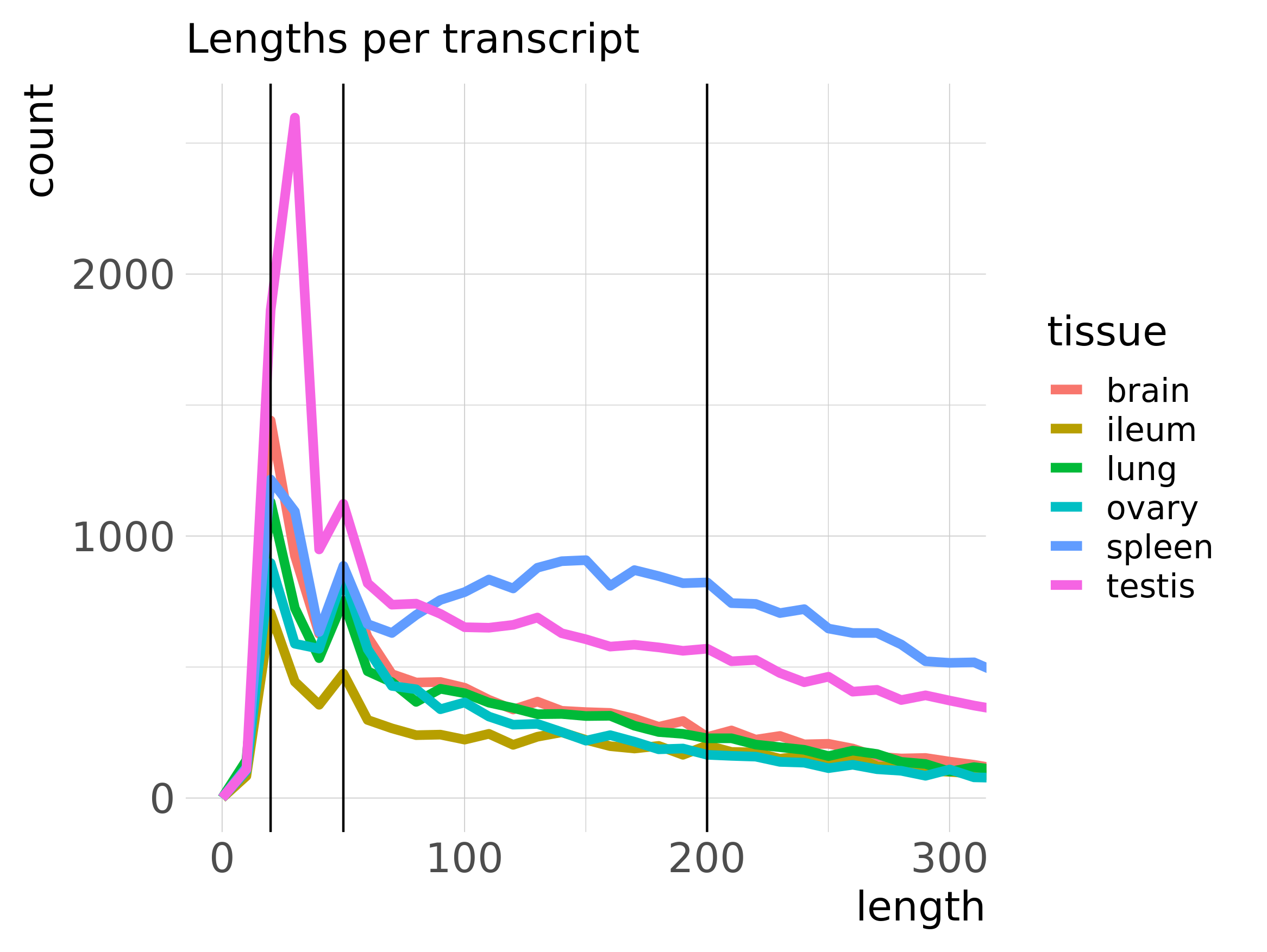

Supplement: giab081_Supplemental_Files [file giab081_supplemental_files.zip › s8_transcript_lengths.png]

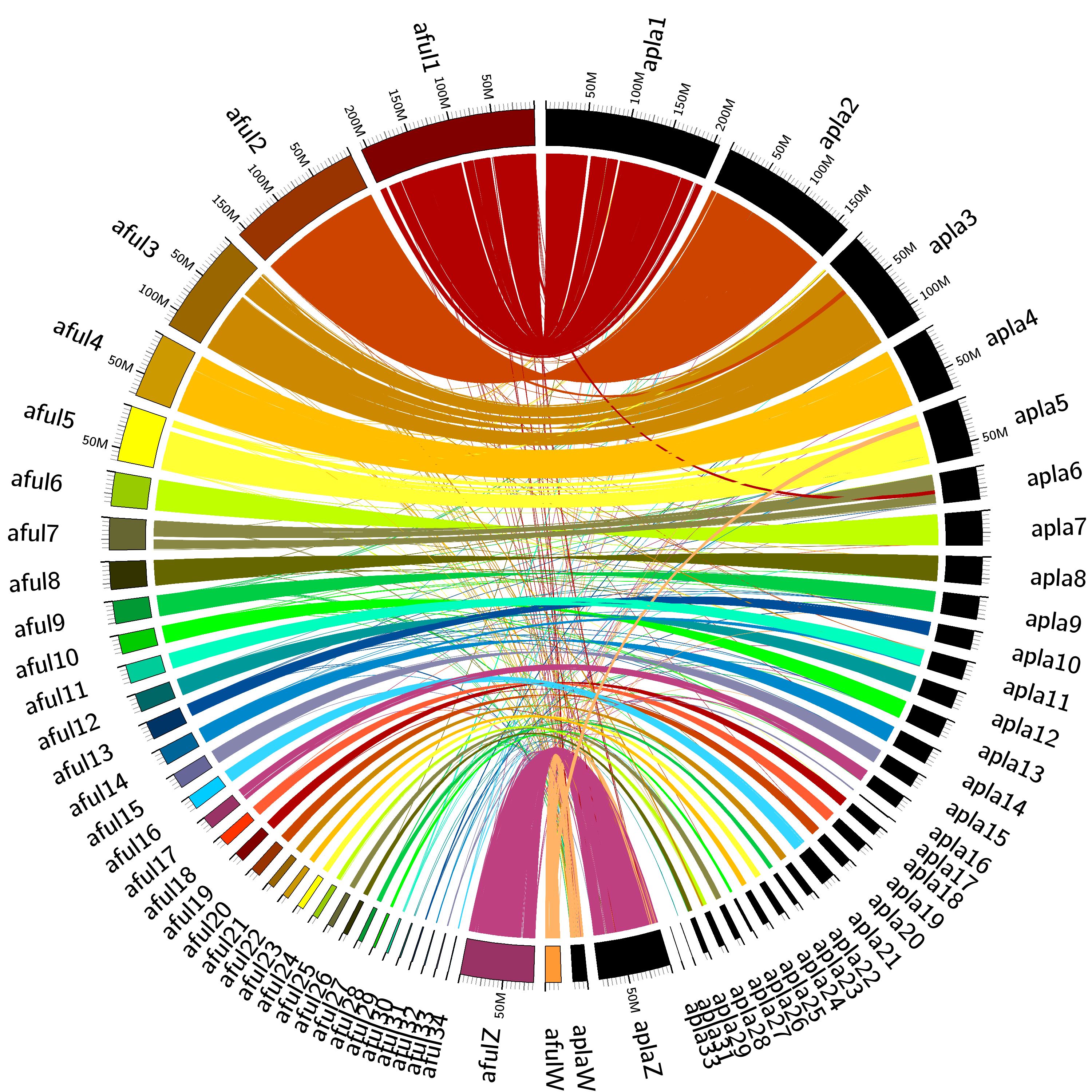

Supplement: giab081_Supplemental_Files [file giab081_supplemental_files.zip › s9_tufted_mallard_synteny.png]
