# Supplementary material for: A high-quality genome and comparison of short- versus long-read transcriptome of the palaearctic duck Aythya fuligula (tufted duck)
Source: Gigascience. 2021 Dec 20;10(12):giab081. doi: 10.1093/gigascience/giab081 (PMC8685854; doi:10.1093/gigascience/giab081)
Supplement: giab081_Response_to_Reviewer_Comments_Original_Submission [file giab081_response_to_reviewer_comments_original_submission.pdf]

Author's Response To Reviewer Comments

Close

Dear Editor,

We hereby submit a revised version of the manuscript "A high-quality Genome and Comparison of Short versus Long Read Transcriptome of the Palaearctic duck Aythya fuligula (Tufted Duck)" by Mueller et al. (manuscript ID: GIGA-D-21-00072).
On behalf of my co-authors, we thank you for the opportunity to revise our manuscript. We are grateful for the reviewers' feedback and have revised our manuscript accordingly. Additions in the manuscript (20210715_rmueller_tufted_duck_diff.pdf) are marked in blue and deleted parts red/strike-through. A point-by-point response is below.

Yours Sincerely,
Ralf C Mueller; Patrik Ellström; Kerstin Howe; Marcela Uliano-Silva; Richard I Kuo; Katarzyna Miedzinska; Amanda Warr; Olivier Fedrigo; Bettina Haase; Jacquelyn Mountcastle; William Chow; James Torrance; Jonathan MD Wood; Josef D Järhult; Mahmoud M Naguib; Björn Olsen; Erich D Jarvis; Jacqueline Smith; Lél Eöry; Robert HS Kraus

Reviewer #1:

1. The introduction is largely devoted to the great advantage of PacBio over Illumina techniques in elucidating the non-model species' genome feature. This is not needed for the authors of Gigascience. I suggest the authors provide more information in the tufted duck. From the previous studies, how diverged in terms of million years and sequence level between the tufted duck vs. mallard? What is the phylogenetic position of tufted duck in Galloanserase? Are there any lab or field studies of tufted duck's susceptibility to AIV? What is the potential genetic cause? Also, since it is known in mallards that RIG-I is responsible for the AVI response, is this gene then intact or how is this gene expressed in the newly presented tufted duck genome?

The introduction has been shortened by a whole paragraph comparing short-read and long-read sequencing technology. Instead, additional background information on the tufted duck has been provided in the introduction's first paragraph. Additionally, an analysis of the presence and expression of the RIG-I/DDX58 gene has been conducted in comparison with mallard, and related information provided in methods, results, discussion and supplements.

2. The analyses part needs to show the repeat content of tufted duck and its comparison to other avian genomes. Particularly, the repeat content of Z and W chromosome. Did the author look into the centromere or telomere sequences?

During genome assembly curation, no telomeres or centromeres were detected. However, we analysed the repeat content and composition in the tufted duck genome using RepeatMasker and RepeatModeler. We further compared the repeat content, both overall and at the chromosome level, between the tufted duck and the mallard genome assemblies. As the main focus of this paper is on the tufted duck transcriptome, we described the method and the results in the main text but added corresponding figures S2-4 to the supplements.

3. Are Z and W chromosomes assembled into two intact sequences? If so, evidence is needed to show that there is no chimeric assembly between Z and W, or other autosome sequences, as it is mentioned in the paper that 'most of the genome separated into haplotypes'.

The Z/W chromosomes were detailed in the first paragraph of the Results section, and an additional figure S1 provided in the supplements.

4. Tissue-specific expression part: Here what does 'supported' gene mean exactly? Just to make sure,

the authors means 'genes' or 'transcripts'?

It is genes, but to (hopefully) make this clearer, the respective methods section 'Tissue-specific expression analysis' was rephrased.

5. 'Stringtie2 may discard single-exon transcript model..' Did the author find that the Stringtie2 results generally have a much lower proportion of single-exon transcripts compared to say, Iso-seq data?

This statement might have been indeed misleading since the argument was based on the exons-per-gene plot. We conducted an extra analysis of exons per transcript described in the respective result section, and figure S5 was added.

6. 'The average number of transcripts in the long-read pipeline often almost matched..': I am confused here that Figure 5 shows the opposite result that the supported PacBio genes are much lower in number than those produced by Illumina reads. Any results to support this claim?

Admittedly, there may have been two issues: The number of genes in figure 5 does not match the number of genes in Table 3. Table 3 shows all reconstructed transcript models, and figure 5 is based on functionally annotated genes (all hits in UniRef50). We tried to clarify this in the discussion, discussing the implication that the transcript model reconstruction's high number in PacBio is not reflected in the functional annotation. We also rephrased the wording from "Annotation" to "Transcript model reconstruction" in the appropriate places to better differentiate between transcript model reconstruction and functional annotation.

7. 'This distribution is much more balanced in the long-read pipeline..' here the authors may suggest that PacBio iso-seq recover more alternative splicing transcripts compared to Illumina data. But it is unclear that the supported genes of iso-seq are so much lower in number than those of Illumina, which may be caused by the relatively lower coverage of iso-seq? So I would conclude at least in this study, both techniques are complementary to each other, rather than one performing better than the other.

We clarified that this analysis is based on all UniRef50-identified hits and corrected the wrong inference on transcripts while talking about genes. The result (figure 5) is indeed most likely because of the lower coverage in IsoSeq (e.g., a relatively high number of genes are supported in individual tissues, just not in all (unlike in the Illumina pipeline)). Also see R2, Q 7-9; we conclude that both technologies were complementary in our study and emphasise the recommendation to use long-read transcriptome reconstruction with higher coverage in the future (mRNA/cDNA for this study was sequenced 2017/2018).

8. How are the TEs annotated by these small RNAs, as apart from miRNAs, there should be large portions of piRNAs mapping to TEs.

No piRNAs were detected in our small RNA analysis.

Reviewer #2:

1. Are the of 34 autosomes + sex chromosomes expected? Was there an a priori expectation based on the karyotype or based on the mallard genome assembly? Was this expectation provided during the scaffolding using HiC?

The karyotype of Aythya fuligula is n=39, as stated in the first paragraph of the results section. Of these, 34 +ZW are all that could confidently be recovered in the curation process. The a priori knowledge is not used in the scaffolding. However, from a manual curation perspective, having the karyotype aids greatly in birds where many chromosomes are very small.

2. Does the assembly size match the expected genome size based on an independent estimate?

The assembly length after curation is close to the expected size of duck genomes. This information has been added in the first paragraph of the results section.

3. Since this is a chromosome-scale assembly, what are the metrics of individual chromosomes? I see in

NCBI that the chromosome numbers have been assigned, is this based on size or homology to chicken chromosomes? What are the lengths of each chromosome? GC content? Gene content? Gaps? How many contigs were scaffolded by HiC to build each chromosome?

The chromosome naming is based on size, and the assembly stats can be taken from NCBI. However, we provided some additional information on the curation in tables S1 and S2.

4. More detail is needed regarding how the manual curation is done so it can be repeatable by other researchers.

The curation pipeline is explained in great detail in Howe et al., 2021 and cited in the methods part; however, it is now also cited in the data description and results.

5. What is the sequencing effort (# lanes, # SMRT cells, etc.) and resulting coverage from each technology of the genome? This doesn't have to be very detailed if it is reported elsewhere but some idea for the reader will be helpful.

The coverage for each applied sequencing method was added, and a reference to the raw data (including description) is provided in the second paragraph of the results section.

6. I am aware that the VGP pipeline was used for the assembly, is there a GitHub for the pipeline that can be included which has the specific commands and flags for each step?

A reference pointing to the VGP assembly pipeline on GitHub has been provided, and the reference to the meanwhile peer-reviewed VGP flagship article Rhie et al., 2021 has been updated and provided at appropriate places within the manuscript.

Since the annotation comparison was exhaustive, I don't have as many comments on it. The authors may not be explicitly making a recommendation on which approach to use but what is a good metric that the readers can use to compare the results considering the orders of magnitude difference in sequencing coverage?

7. Regarding the Illumina and PacBio annotation comparisons, since the coverage are substantially different, in what metric are they comparable? Is it similar in sequencing cost?

Sequencing costs are permanently changing and thus difficult to compare or to give a recommendation based on that. It would not make much sense to report "our" sequencing costs since sequencing took place in 2017 and 2018.

8. Would the PacBio still underperform in terms of recovered genes if it had the same coverage as the Illumina libraries?

In part, this is answered with an updated discussion of tissue-specific expression, and a recommendation for long-read transcriptome sequencing with high coverage has been emphasised in the conclusions.

9. What was the sequencing effort for the PacBio IsoSeq?

We are not entirely sure what is meant by "effort" in this context; if it relates to costs or sequencing duration, please see R2/7.

Close
