# Supplementary material for: A high-quality genome and comparison of short- versus long-read transcriptome of the palaearctic duck Aythya fuligula (tufted duck)
Source: Gigascience. 2021 Dec 20;10(12):giab081. doi: 10.1093/gigascience/giab081 (PMC8685854; doi:10.1093/gigascience/giab081)
Supplement: giab081_Response_to_Reviewer_Comments_Revision_1 [file giab081_response_to_reviewer_comments_revision_1.pdf]

Author's Response To Reviewer Comments

Close

Dear Editor,

We hereby submit a revised version of the manuscript "A high-quality Genome and Comparison of Short versus Long Read Transcriptome of the Palaearctic duck Aythya fuligula (Tufted Duck)" by Mueller et al. (manuscript ID: GIGA-D-21-00072).
On behalf of my co-authors, we thank you for the opportunity to revise our manuscript. We are grateful for the reviewers' feedback and have revised our manuscript accordingly. Additions in the manuscript's supplementary material are marked in blue and deleted parts red/strike-through (20211012_rmueller_tufted_duck_supp_diff.pdf). A point-by-point response is below.

Yours Sincerely,
Ralf C Mueller; Patrik Ellström; Kerstin Howe; Marcela Uliano-Silva; Richard I Kuo; Katarzyna Miedzinska; Amanda Warr; Olivier Fedrigo; Bettina Haase; Jacquelyn Mountcastle; William Chow; James Torrance; Jonathan MD Wood; Josef D Järhult; Mahmoud M Naguib; Björn Olsen; Erich D Jarvis; Jacqueline Smith; Lél Eöry; Robert HS Kraus

Reviewer #1: For question 3:

"The author need to explain more about why they think Figure S1 shows there is no chimeric assembly between Z and W chromosome. As Figure S1 is just a Hi-C matrix plot, among the submitted materials, I also cannot find the legend explaining the figure."

Thank you for your feedback! Figure S1 has been re-worked, including scales, labels for the Z/W chromosomes, and a more detailed legend (20211012_rmueller_tufted_duck_supp.pdf). The Hi-C contact map in figure S1 shows the near-complete separation of the Z and W chromosomes, except for the 2.1 Mb pseudoautosomal region. We do not understand why reviewer #1 could not find the legend in our previously submitted version and therefore seize the opportunity also to add the updated legend text here:
"Hi-C contact map of Aythya fuligula visualised in HiGlass. The plot shows the interaction in the chromatin space of chromosomes Z and W. The shading indicates the level of interaction occurring along the chromosomes: the more closely interacting, the greater the degree of shading. This Hi-C map shows the near-complete separation of the sex chromosomes, except for the contact interaction in the 2.1 Mb pseudoautosomal region (black box), for which there is only a single representation in the assembly of the Z chromosome."

Close
