# Supplementary material for: A high-quality genome and comparison of short- versus long-read transcriptome of the palaearctic duck Aythya fuligula (tufted duck)
Source: Gigascience. 2021 Dec 20;10(12):giab081. doi: 10.1093/gigascience/giab081 (PMC8685854; doi:10.1093/gigascience/giab081)
Supplement: giab081_Response_to_Reviewer_Comments_Revision_2 [file giab081_response_to_reviewer_comments_revision_2.pdf]

Author's Response To Reviewer Comments

Close

Dear Editor,

We hereby submit a revised version of the manuscript "A high-quality Genome and Comparison of Short versus Long Read Transcriptome of the Palaearctic duck Aythya fuligula (Tufted Duck)" by Mueller et al. (manuscript ID: GIGA-D-21-00072).
On behalf of my co-authors, we thank you for the opportunity to revise our manuscript. We are grateful for the reviewers' feedback and have revised our manuscript accordingly.

Yours Sincerely,
Ralf C Mueller; Patrik Ellström; Kerstin Howe; Marcela Uliano-Silva; Richard I Kuo; Katarzyna Miedzinska; Amanda Warr; Olivier Fedrigo; Bettina Haase; Jacquelyn Mountcastle; William Chow; James Torrance; Jonathan MD Wood; Josef D Järhult; Mahmoud M Naguib; Björn Olsen; Erich D Jarvis; Jacqueline Smith; Lél Eöry; Robert HS Kraus

Close
