# Supplementary material for: A high-quality genome and comparison of short- versus long-read transcriptome of the palaearctic duck Aythya fuligula (tufted duck)
Source: Gigascience. 2021 Dec 20;10(12):giab081. doi: 10.1093/gigascience/giab081 (PMC8685854; doi:10.1093/gigascience/giab081)
Supplement: giab081_Reviewer_1_Report_Original_Submission — Qi Zhou -- 4/7/2021 Reviewed [file giab081_reviewer_1_report_original_submission.pdf]

Reviewer Report

Title: A high-quality Genome and Comparison of Short versus Long Read Transcriptome of the Palaearctic duck Aythya fuligula (Tufted Duck)

Version: Original Submission **Date:** 4/7/2021

Reviewer name: Qi Zhou

Reviewer Comments to Author:

Dear Editor:
Mueller et al. presented a high-quality genome and annotation of tufted duck with combined long-read and short-read techniques here. Tufted duck shows a different susceptibility to avian influenza A viruses (AIV) compared to mallards that share the habitat. So besides adding a new avian genomic resource, tufted duck genome may facilitate the research into the genetic basis of AIV infection. Overall, I think the genome is of high quality, but I do have several comments below:

1. The introduction is largely devoted to the great advantage of PacBio over Illumina techniques in elucidating the non-model species' genome feature. This is not needed for the authors of Gigascience. I suggest the authors provide more information in the tufted duck. From the previous studies, how diverged in terms of million years and sequence level between the tufted duck vs. mallard? What is the phylogenetic position of tufted duck in Galloanserase? Are there any lab or field studies of tufted duck's susceptibility to AIV? What is the potential genetic cause? Also, since it is known in mallards that RIG-I is responsible for the AVI response, is this gene then intact or how is this gene expressed in the newly presented tufted duck genome?
2. The analyses part needs to show the repeat content of tufted duck and its comparison to other avian genomes. Particularly, the repeat content of Z and W chromosome. Did the author look into the centromere or telomere sequences?
3. Are Z and W chromosomes assembled into two intact sequences? If so, evidence is needed to show that there is no chimeric assembly between Z and W, or other autosome sequences, as it is mentioned in the paper that 'most of the genome separated into haplotypes'.
4. Tissue-specific expression part: Here what does 'supported' gene mean exactly? Just to make sure, the authors means 'genes' or 'transcripts'?
5. 'Stringtie2 may discard single-exon transcript model..' Did the author find that the Stringtie2 results generally have a much lower proportion of single-exon transcripts compared to say, Iso-seq data?
6. 'The average number of transcripts in the long-read pipeline often almost matched..': I am confused here that Figure 5 shows the opposite result that the supported PacBio genes are much lower in number than those produced by Illumina reads. Any results to support this claim?
7. 'This distribution is much more balanced in the long-read pipeline..' here the authors may suggest that PacBio iso-seq recover more alternative splicing transcripts compared to Illumina data. But it is unclear that the supported genes of iso-seq are so much lower in number than those of Illumina, which may be caused by the relatively lower coverage of iso-seq? So I would conclude at least in this study, both techniques are complementary to each other, rather than one performing better than the other.

8. How are the TEs annotated by these small RNAs, as apart from miRNAs, there should be large portions of piRNAs mapping to TEs.

Level of Interest

Please indicate how interesting you found the manuscript: Choose an item.

Quality of Written English

Please indicate the quality of language in the manuscript: Choose an item.

Declaration of Competing Interests

Please complete a declaration of competing interests, considering the following questions:

- Have you in the past five years received reimbursements, fees, funding, or salary from an organisation that may in any way gain or lose financially from the publication of this manuscript, either now or in the future?
- Do you hold any stocks or shares in an organisation that may in any way gain or lose financially from the publication of this manuscript, either now or in the future?
- Do you hold or are you currently applying for any patents relating to the content of the manuscript?
- Have you received reimbursements, fees, funding, or salary from an organization that holds or has applied for patents relating to the content of the manuscript?
- Do you have any other financial competing interests?
- Do you have any non-financial competing interests in relation to this paper?

If you can answer no to all of the above, write 'I declare that I have no competing interests' below. If your reply is yes to any, please give details below.

I declare that I have no competing interests

I agree to the open peer review policy of the journal. I understand that my name will be included on my report to the authors and, if the manuscript is accepted for publication, my named report including any attachments I upload will be posted on the website along with the authors' responses. I agree for my report to be made available under an Open Access Creative Commons CC-BY license (http://creativecommons.org/licenses/by/4.0/). I understand that any comments which I do not wish to be included in my named report can be included as confidential comments to the editors, which will not be published.

Choose an item.

To further support our reviewers, we have joined with Publons, where you can gain additional credit to further highlight your hard work (see: https://publons.com/journal/530/gigascience). On publication of this paper, your review will be automatically added to Publons, you can then choose whether or not to claim your Publons credit. I understand this statement.

Yes Choose an item.
