# Supplementary material for: A high-quality genome and comparison of short- versus long-read transcriptome of the palaearctic duck Aythya fuligula (tufted duck)
Source: Gigascience. 2021 Dec 20;10(12):giab081. doi: 10.1093/gigascience/giab081 (PMC8685854; doi:10.1093/gigascience/giab081)
Supplement: giab081_Reviewer_2_Report_Original_Submission — Joshua PeÃ±alba -- 4/23/2021 Reviewed [file giab081_reviewer_2_report_original_submission.pdf]

Reviewer Report

Title: A high-quality Genome and Comparison of Short versus Long Read Transcriptome of the Palaearctic duck Aythya fuligula (Tufted Duck)

Version: Original Submission **Date:** 4/23/2021

Reviewer name: Joshua PeÃ±alba

Reviewer Comments to Author:

This data note by Mueller et al. describes the high-quality, chromosome-scale assembly of the tufted duck and the gene annotation using both Illumina and PacBio sequencing. The authors present and compare the resulting annotations from the different sequencing platforms which is useful for researchers intending to do RNAseq for annotation.
I think the details in the note focuses primarily on the gene annotation comparison and the genome assembly has not received adequate attention. Since this will likely be the data note that reports on the genome assembly, it should probably have more details on the chromosomes (in detail below). I understand that one can go into an exhaustive description but I think these as a minimum will give the reader a good idea about the quality of the genome assembly:
- Are the of 34 autosomes + sex chromosomes expected? Was there an a priori expectation based on the karyotype or based on the mallard genome assembly? Was this expectation provided during the scaffolding using HiC?
- Does the assembly size match the expected genome size based on an independent estimate?
- Since this is a chromosome-scale assembly, what are the metrics of individual chromosomes? I see in NCBI that the chromosome numbers have been assigned, is this based on size or homology to chicken chromosomes? What are the lengths of each chromosome? GC content? Gene content? Gaps? How many contigs were scaffolded by HiC to build each chromosome?
- More detail is needed regarding how the manual curation is done so it can be repeatable by other researchers.
- What is the sequencing effort (# lanes, # SMRT cells, etc.) and resulting coverage from each technology of the genome? This doesn't have to be very detailed if it is reported elsewhere but some idea for the reader will be helpful.
- I am aware that the VGP pipeline was used for the assembly, is there a GitHub for the pipeline that can be included which has the specific commands and flags for each step?
Since the annotation comparison was exhaustive, I don't have as many comments on it. The authors may not be explicitly making a recommendation on which approach to use but what is a good metric that the readers can use to compare the results considering the orders of magnitude difference in sequencing coverage?
- Regarding the Illumina and PacBio annotation comparisons, since the coverage are substantially different, in what metric are they comparable? Is it similar in sequencing cost?
- Would the PacBio still underperform in terms of recovered genes if it had the same coverage as the Illumina libraries?

- What was the sequencing effort for the PacBio IsoSeq?

Level of Interest

Please indicate how interesting you found the manuscript: Choose an item.

Quality of Written English

Please indicate the quality of language in the manuscript: Choose an item.

Declaration of Competing Interests

Please complete a declaration of competing interests, considering the following questions:

- Have you in the past five years received reimbursements, fees, funding, or salary from an organisation that may in any way gain or lose financially from the publication of this manuscript, either now or in the future?
- Do you hold any stocks or shares in an organisation that may in any way gain or lose financially from the publication of this manuscript, either now or in the future?
- Do you hold or are you currently applying for any patents relating to the content of the manuscript?
- Have you received reimbursements, fees, funding, or salary from an organization that holds or has applied for patents relating to the content of the manuscript?
- Do you have any other financial competing interests?
- Do you have any non-financial competing interests in relation to this paper?

If you can answer no to all of the above, write 'I declare that I have no competing interests' below. If your reply is yes to any, please give details below.

I declare that I have no competing interests.

I agree to the open peer review policy of the journal. I understand that my name will be included on my report to the authors and, if the manuscript is accepted for publication, my named report including any attachments I upload will be posted on the website along with the authors' responses. I agree for my report to be made available under an Open Access Creative Commons CC-BY license (http://creativecommons.org/licenses/by/4.0/). I understand that any comments which I do not wish to be included in my named report can be included as confidential comments to the editors, which will not be published.

Choose an item.

To further support our reviewers, we have joined with Publons, where you can gain additional credit to further highlight your hard work (see: https://publons.com/journal/530/gigascience). On publication of this paper, your review will be automatically added to Publons, you can then choose whether or not to claim your Publons credit. I understand this statement.

Yes Choose an item.
